# A numerical and analytical evaluation of the consistency of the energy balance model identifies limitations and systematic relationships between mass intake and body weight dynamics

**DOI:** 10.3389/fnut.2026.1777554

**Published:** 2026-07-30

**Authors:** Francisco Arencibia-Albite

**Affiliations:** School of Health and Sciences, Universidad del Sagrado Corazón, San Juan, Puerto Rico

**Keywords:** body composition, body weight, energy balance model, mass balance, numerical consistency

## Abstract

The Energy Balance Model (EBM) is widely used to interpret body weight regulation by relating changes in body mass to the imbalance between energy intake and energy expenditure. Despite its central role in nutritional science, the empirical reliability and internal consistency of this framework have rarely been evaluated using independently measured physiological quantities obtained under controlled experimental conditions. The present study examines both the numerical and analytical coherence of the EBM. First, three numerical consistency tests derived from the governing accounting relationships of the EBM are introduced. These tests evaluate whether independently measured variables defining energy balance and macronutrient balance yield mutually compatible predictions when applied to metabolic-ward datasets. The tests are applied to two highly influential randomized controlled trials in which energy intake, energy expenditure, and body composition were measured under tightly controlled inpatient conditions. In parallel, the analysis examines whether total food mass intake exhibits a more systematic correspondence with observed changes in body weight and body composition. The results indicate that the correspondence between measured energy imbalance and observed body mass change is weaker than expected from a quantitatively predictive framework. In contrast, variation in food mass intake shows a more consistent association with changes in body weight under the same experimental conditions. In addition, analytical examination of the EBM accounting structure shows that when body weight stability is interpreted as energy balance, and macronutrient fluxes and obligatory protein loss are explicitly accounted for, the model implies an internally inconsistent physiological state: a persistent negative protein balance despite stable body weight. These findings highlight limitations in the predictive and internal consistency of the energy balance framework and support the need for frameworks that analyze body weight dynamics explicitly in terms of material balance and appetite-regulated mass intake.

## 1 Introduction

Quantitative scientific models are expected to satisfy two fundamental conditions: their governing equations must remain consistent with empirical observations, and their input variables must be measurable with sufficient precision to permit reproducible predictions (1–3). In computational physiology, these standards are routinely achieved, reflecting that the governing equations capture, at least approximately, the dominant causal structure underlying the system under investigation (4, 5).

By contrast, the Energy Balance Model (EBM), long regarded as a quantitative framework for human body weight regulation, has exhibited variable predictive performance when tested against empirical data, contributing to increased interest in alternative modeling paradigms aimed at improving predictive consistency and physiological plausibility (6, 7). Despite its apparent simplicity, the empirical validity of the model has often been assumed rather than explicitly demonstrated, as reflected in the wide diversity of distinct EBM implementations reported in the literature (8–24). Consequently, if the EBM is to be considered as a quantitatively predictive physiological model, it must exhibit numerical fidelity comparable to that of established models, yielding small, reproducible relative errors under well-controlled experimental conditions. In this regard, the present analysis does not evaluate a simplified or hypothetical representation of the EBM but rather examines the numerical implications of the same accounting relationships that are routinely applied in metabolic studies of body weight regulation, including the metabolic-ward experiments analyzed in this work.

In practice, evaluations of the EBM tend to attribute discrepancies between prediction and measurement to experimental imperfections such as participant underreporting, instrument error, or methodological variability rather than to structural limitations of the theory itself (25–28). As an illustration of these issues, during isocaloric feeding trials designed to test the effects of diet composition on body weight change, researchers frequently report no statistically significant differences in energy imbalance between diet groups, yet the observed weight loss outcomes differ significantly (26, 27, 29, 30). From a quantitative standpoint, such results appear difficult to reconcile with the governing equation of the EBM, which posits that body mass change depends ultimately on the imbalance between energy intake and energy expenditure. These empirical discrepancies therefore raise concerns regarding the internal consistency of the model and its capacity to function as a physically grounded mechanistic theory.

Advocates of the framework commonly argue that these failures arise from suboptimal experimental control, maintaining that only tightly controlled randomized inpatient metabolic-ward studies can provide the precision necessary to confirm the model's validity (31–34). While this defense initially appears reasonable, it effectively shifts the explanatory burden from theory to context: rather than questioning whether the model itself can accommodate real-world variability, it assumes that deviations from prediction must reflect inadequate experimental conditions. Yet if a model's correctness depends on nearly ideal laboratory environments, its generalizability and quantitative robustness are, by definition, limited.

Although discrepancies between predicted and observed weight change under the energy balance framework have been recognized previously, most discussions have focused on improving empirical predictions by incorporating additional physiological mechanisms, such as metabolic adaptation (e.g., adaptive thermogenesis) (35–37). While these approaches address potential sources of prediction error, they do not directly examine whether the governing accounting relationships of the EBM remain numerically and logically self-consistent when applied to independently measured physiological quantities. Explicit tests evaluating whether the accounting equations underlying the EBM remain numerically self-consistent when applied to independently measured physiological variables have not previously been reported.

The present article addresses this issue by evaluating the numerical consistency of the EBM using data obtained under tightly controlled metabolic-ward conditions and its analytical consistency through mathematical analysis. Specifically, the study introduces a set of numerical consistency tests designed to determine whether independent estimates of energy balance and macronutrient balance yield mutually compatible predictions when applied to experimentally measured quantities. These tests are applied to two landmark randomized controlled trials conducted by Hall and colleagues, in which energy intake, energy expenditure, and body composition were measured under tightly controlled inpatient conditions (38, 39). In parallel, the analysis examines whether alternative measurable quantities, particularly total food mass intake, exhibit a more systematic correspondence with observed changes in body weight and body composition. This comparison provides an empirical basis for evaluating whether body mass dynamics are more consistently described in terms of energy balance or material balance. In addition to this empirical analysis, the study also examines the internal analytical consistency of the EBM accounting framework itself, evaluating whether the commonly invoked coincidence of energy balance and body weight stability leads to logically consistent outcomes when macronutrient fluxes and obligatory protein loss are considered explicitly.

In this context, it is important to distinguish between the incorporation of physiological processes within EBM formulations and the physical principle that ultimately closes these models (34). Contemporary EBM implementations include physiological dynamics, such as adaptive thermogenesis or changes in substrate oxidation, and represent these processes explicitly within their internal structure (19). However, regardless of the level of physiological detail, all EBM variants ultimately relate changes in body mass to the same physical closure condition, namely that the imbalance between energy intake and energy expenditure equals the rate of change of stored body energy (8–24). The present analysis does not question the relevance of physiology in body weight regulation, but rather examines whether this shared energetic closure provides a physically and numerically consistent basis for quantitative prediction.

Finally, the metabolic-ward data reported by Hall et al. (39) are treated as high-quality empirical observations collected under experimental conditions designed to meet the methodological standards typically invoked in support of the EBM. The analysis therefore evaluates both the numerical predictive performance of the EBM with respect to these data and the internal analytical consistency of the framework itself. In doing so, the study provides a systematic evaluation of the EBM that combines empirical numerical consistency tests with an analytical examination of the internal accounting structure of the model, thereby assessing both its predictive reliability and its theoretical coherence.

## 2 Manuscript structure and analytical transparency

To guide the reader through the logical organization of this article, the structure of the manuscript is briefly outlined. First, three numerical consistency tests derived from the governing principles of the EBM are established. Second, these tests are applied to metabolic-ward data from two randomized controlled trials conducted by Hall et al. (38, 39) to evaluate whether the model accurately predicts both the direction and magnitude of body weight change under tightly controlled experimental conditions. Third, the analysis examines whether alternative measurable quantities within the dataset of Hall et al. (39), particularly total food mass intake, exhibit systematic relationships with observed changes in body weight and body composition, and assesses whether these relationships are more consistent with a mass balance interpretation of body weight dynamics (40, 41). Fourth, theoretical constraints that arise when energy balance and weight stability are assumed to coincide are examined, and their implications for the internal logical coherence of the EBM are analyzed.

To preserve clarity and accessibility, complete derivations of all equations and details of the analytical proofs omitted from the main text are provided in the Supplementary material, ensuring that no step in the argument depends on unstated assumptions or unexplained transformations. This allows readers to evaluate the mathematical foundations independently and verify that the results follow from first principles rather than from *ad hoc* or implicit assumptions. Descriptive data are reported as mean values with their associated standard deviation (mean ± SD), unless otherwise specified. Where inferential statistical tests are applied, statistical significance is defined as P < 0.05. One-tailed tests are used when the comparison under consideration involved a clear directional expectation, whereas two-tailed tests are used when departures in either direction are considered relevant.

Because the numerical tests involve comparisons between measured metabolic variables and model predictions, potential sources of physiological and methodological variability must be considered when interpreting the results. In particular, short-term dietary interventions may involve transient physiological changes such as glycogen depletion, associated water shifts, and metabolic adaptation (19, 42, 43). Similarly, measurements of energy intake, energy expenditure, and body composition are subject to known methodological uncertainties (38, 44, 45). These factors are therefore explicitly considered in the interpretation of the numerical results. At the same time, the analysis evaluates the same accounting relationships that are commonly used in energy balance formulations in the metabolic literature, including those applied in the metabolic-ward studies examined here. The purpose of the numerical tests is therefore not to construct a simplified theoretical model, but to assess whether the quantitative relationships implied by the EBM remain internally consistent and empirically predictive when applied to controlled experimental datasets.

### 2.1 A first numerical consistency test for the EBM

Contemporary discussions of the EBM frequently assume, without formal quantitative derivation, that the difference between daily energy intake, *EI* (kcal/day), and daily energy expenditure, *EE* (kcal/day), equals the daily change in total body energy content (34). When this qualitative statement is translated into precise mathematical form, it is represented as


$$\begin{array}{l} EI-EE=\rho_{FM}\frac{dFM}{dt}+\rho_{FFM}\frac{dFFM}{dt} \end{array}$$ (1)

where *FM* and *FFM* denote fat mass and fat-free mass, respectively; ρ_*X*_ represents the energy density (kcal/kg) of body compartment *X*; and *t* is time in days (20, 46).

Equation 1 is EBM's governing equation and expresses the actual value of the daily energy balance, since any caloric imbalance must manifest as measurable changes in the energy content of *FM* and *FFM*. In its application to experimental data, Equation 1 is integrated over a time interval of duration *T* to compute the average energy balance, 〈*EI*〉−〈*EE*〉, during that period (38):


$$\begin{array}{l} \langle EI\rangle -\langle EE\rangle =\frac{\rho_{FM}\text{Δ}FM+\rho_{FFM}\text{Δ}FFM}{T}\\ \;=\frac{9,300\frac{\text{kcal}}{\text{kg}}\times \text{Δ}FM+1,100\frac{\text{kcal}}{\text{kg}}\times \text{Δ}FFM}{T} \end{array}$$ (2)

where the angular brackets indicate the average value of *X*, Δ indicates the net change over *T*, and ρ_*FM*_ = 9,300 kcal/kg (38), ρ_*FFM*_ = 1,100 kcal/kg (38).

For the EBM to be internally consistent as a quantitative framework, the average energy balance computed from Equation 2 must yield values comparable to those obtained through direct empirical assessment, specifically by subtracting the average energy expenditure measured by indirect or direct calorimetry from the average energy intake corrected for unabsorbed energy. Any substantial divergence between these independently derived estimates would indicate a numerical inconsistency within the EBM formulation and would undermine its empirical validity. This equivalence is not an optional property but a necessary consequence of adopting Equation 1 as the governing equation and therefore functions as a mandatory numerical consistency test for the EBM.

### 2.2 The second version of EBM governing equation leads to a second test for numerical consistency

Implicit in the EBM governing equation (Equation 1) is the prediction that if two individuals with similar body mass energy densities experience equivalent energy imbalances, they should undergo comparable changes in body weight (BW). Making this implication explicit leads to an alternative but mathematically equivalent formulation of the governing equation. This reformulation clarifies the central claim of the EBM, namely, that energy imbalance, regardless of its origin, determines the rate of body weight change.

To derive this result, note that it is always the case that


$$\begin{array}{l} \frac{dBW}{dt}=\frac{dFM}{dt}+\frac{dFFM}{dt} \end{array}$$

which can be rearranged into two equivalent forms:


$$\begin{array}{l} \frac{dFM}{dt}=\left[\frac{1}{1+dFFM/dFM}\right]\frac{dBW}{dt},\\ \frac{dFFM}{dt}=\left[\frac{dFFM/dFM}{1+dFFM/dFM}\right]\frac{dBW}{dt} \end{array}$$

Substituting these expressions into Equation 1 and solving for *dBW/dt* yields


$$\begin{array}{l} \frac{dBW}{dt}=\frac{EI-EE}{\rho_{BM}} \end{array}$$ (3)

where ρ_*BM*_ is the body mass energy density defined as


$$\begin{array}{l} \rho_{BM}=\frac{\rho_{FM}+\rho_{FFM}dFFM/dFM}{1+dFFM/dFM} \end{array}$$

which, as required by the EBM, varies as body composition changes (47, 48).

Equation 3 is therefore mathematically equivalent to the original governing equation of the EBM (Equation 1), but makes explicit the dependence of body weight change on energy imbalance scaled by body mass energy density. This formulation implies that individuals with similar values of ρ_*BM*_ should exhibit comparable magnitudes of weight change when subjected to similar energy imbalances. Accordingly, Equation 3 establishes a second criterion for assessing the numerical consistency of the EBM. Under rigorously controlled randomized conditions, where subjects have comparable body mass energy densities and experience similar levels of energy imbalance, the model predicts that no statistically significant differences in weight change should be observed between groups.

It is important to emphasize that Equation 3 applies to any finite time interval and does not assume that body composition remains constant over that interval. The body mass energy density (ρ_*BM*_) is a time-dependent quantity that can vary as a function of changes in fat mass, fat-free mass, glycogen stores, and associated body water (47, 48). Consequently, transient physiological processes that alter body composition, such as glycogen depletion and fluid shifts, are implicitly incorporated through variations in ρ_*BM*_. The equation therefore remains applicable under both short-term and long-term conditions, provided that all quantities are evaluated over a consistent time interval.

### 2.3 A third numerical consistency test for the EBM

Within the EBM, the temporal evolution of fat mass is governed by the following accounting identity: at all times and under all conditions, the rate of change of fat mass, *dFM/dt*, must equal the difference between the daily absorbed fat intake (*FI*_*a*_, g/day) and the net rate of fat oxidation (*FOx*_*net*_, g/day) (49):


$$\begin{array}{l} \frac{dFM}{dt}=FI_{a}-FOx_{net} \end{array}$$ (4)

Net fat oxidation is defined as total fat oxidation minus the rate of *de novo* lipogenesis, since newly synthesized lipid must be subtracted from oxidative fat loss to obtain the effective depletion of the fat mass compartment (49, 50). Equation 4 has played a central role in the investigation of how diet composition influences body adiposity by linking dietary fat intake and fat oxidation to changes in fat mass (49).

Additionally, by Equation 4 the average daily fat balance, over a period of length *T*, can be expressed as:


$$\begin{array}{l} \langle FI_{a}\rangle -\langle FOx_{net}\rangle =\frac{\text{Δ}FM}{T} \end{array}$$

Therefore, according to the EBM, the change in fat mass, Δ*FM*, over an intervention of duration *T* must equal the product of the average daily fat balance and *T*:


$$\begin{array}{l} \text{Δ}FM=\left[\langle FI\rangle -\langle FOx_{net}\rangle \right]\cdot T \end{array}$$ (5)

where *FI*_*a*_ has been replaced by fat intake (*FI*, g/day), because in the empirical application of Equation 4 the difference between absorbed fat intake and measured fat intake is negligible (49). This relationship is not a heuristic approximation but a required quantitative consequence of the EBM framework. Hence, any empirical deviation from Equation 5 constitutes a numerical inconsistency within the EBM itself, rather than a mere measurement discrepancy. This identity therefore provides a third, independent numerical test of the internal coherence of the EBM.

### 2.4 Results of the first numerical consistency test

Testing the validity of the EBM requires experimental conditions in which energy intake, energy expenditure, and body composition are measured with high fidelity. Such conditions are most closely approximated in tightly controlled metabolic studies conducted under inpatient or metabolic-ward settings. This section reassesses key results from one such randomized controlled trial (38) in order to examine whether the quantitative relationships implied by the EBM remain numerically consistent when applied to high-quality experimental data.

A necessary numerical consequence of the EBM is that the average energy balance computed from Equation 2 using body composition changes must agree with the average energy balance computed from separately measured absorbed energy intake and energy expenditure. This agreement is not merely an empirical tendency but a logical requirement of the energy balance accounting framework: any persistent imbalance between energy intake and energy expenditure must ultimately be reflected in a corresponding change in the energy stored within body tissues. To evaluate this numerical consistency requirement, relative error (RE) is used as the metric, defined as:


$$\begin{array}{l} \text{RE}=|\frac{\left(\text{calculated value}\right)-\left(\text{reference value}\right)}{\text{reference value}}|\times 100. \end{array}$$

where the reference value is that given by Equation 2, since any sustained state of energy imbalance must ultimately produce changes in the energy content of fat mass and fat-free mass. Because relative error can become difficult to interpret when the reference value is small or changes sign, absolute error (AE) was also calculated as:


$$\begin{array}{l} \text{AE}=|\left(\text{calculated value}\right)-\left(\text{reference value}\right)|. \end{array}$$

For a single prediction–observation comparison, the root mean square error is mathematically equivalent to the absolute error. Accordingly, absolute error provides a complementary measure of discrepancy that remains interpretable even when the reference value is small or negative. These metrics are used here only as diagnostic indicators of numerical agreement between independent estimates of energy balance rather than as formal statistical tests.

A relevant case is the randomized controlled trial by Hall et al. (38), which compared unrestricted consumption of ultra-processed and unprocessed diets over a 14-day period, with body composition changes assessed using dual-energy X-ray absorptiometry (DXA). In the ultra-processed arm, participants gained on average 0.4 kg of fat mass and 0.5 kg of fat-free mass, which by Equation 2 corresponds to an average positive energy balance of


$$\begin{array}{r} \langle EI\rangle -\langle EE\rangle =\frac{9,300\frac{\text{kcal}}{\text{kg}}\left(\text{+0 .4kg}\right)+1,100\frac{\text{kcal}}{\text{kg}}\left(\text{+0 .5kg}\right)}{14\text{ days}}\\ =+305\frac{\text{kcal}}{\text{day}} \end{array}$$

Conversely, in the unprocessed arm, fat mass decreased by 0.3 kg and fat-free mass by 0.6 kg, implying a negative average energy balance of


$$\begin{array}{r} \langle EI\rangle -\langle EE\rangle =\frac{9,300\frac{\text{kcal}}{\text{kg}}\left(-\text{0 .3kg}\right)+1,100\frac{\text{kcal}}{\text{kg}}\left(-\text{0 .6kg}\right)}{14\text{ days}}\\ \approx -246\frac{\text{kcal}}{\text{day}}. \end{array}$$

In contrast, when the average energy balance is computed as absorbed intake minus expenditure, using *EI* corrected for unabsorbed energy (approximately 7 percent during overfeeding and 11 percent during underfeeding (51)) and *EE* measured by doubly labeled water, the ultra-processed diet yields an average positive energy balance of


$$\begin{array}{l} \langle 0.93\times EI\rangle -\langle EE\rangle =0.93\times 2,963\frac{\text{kcal}}{\text{day}}-2,546\frac{\text{kcal}}{\text{day}}\approx +210\frac{\text{kcal}}{\text{day}} \end{array}$$

and the unprocessed diet yields an average negative energy balance of


$$\begin{array}{l} \langle 0.89\times EI\rangle -\langle EE\rangle =0.89\times 2,491\frac{\text{kcal}}{\text{day}}-2,375\frac{\text{kcal}}{\text{day}}\approx -158\frac{\text{kcal}}{\text{day}}. \end{array}$$

The corresponding absolute and relative errors are:

Ultra-processed diet:

*AE* = |210 − 305| = 95kcal/day;

$RE=|\frac{210-305}{305}|\times 100\approx 31.1\%$,

Unprocessed diet:

*AE* = |−158−(−246)| = 88kcal/day;

$RE=|\frac{-158-\left(-246\right)}{-246}|\times 100\approx 35.8\%$.

Because discrepancies between independent estimates of energy balance must be interpreted in light of methodological uncertainty, it is important to assess whether the observed differences exceed plausible combined measurement error. For the intake minus expenditure estimate, uncertainties of approximately ±100 to ±150 kcal/day for energy intake and ±100 to ±200 kcal/day for energy expenditure were considered (38, 44). Propagating these uncertainties yields a combined uncertainty of approximately 141–250 kcal/day for the intake-expenditure estimate.

For the body-composition-based estimate, uncertainty propagation was performed using the energy densities ρ_FM_ = 9,300 kcal/kg and ρ_FFM_ = 1,100 kcal/kg (38) and assuming plausible DXA uncertainties of ±0.5 kg for both fat mass and fat-free mass over the 14-day interval (45). This yields an estimated uncertainty of approximately 334 kcal/day. Combining these sources of uncertainty produces a total propagated uncertainty of approximately 362–417 kcal/day. Under a more conservative assumption of ±1.0 kg DXA uncertainty, the combined uncertainty increases to approximately 684–714 kcal/day.

Under either assumption, the observed discrepancies of 95 kcal/day and 88 kcal/day fall within plausible combined methodological uncertainty. Accordingly, the results of this first numerical comparison should be interpreted cautiously. Rather than constituting decisive evidence against the EBM on their own, these results illustrate the limited quantitative agreement that can arise between independent estimates of energy balance even under tightly controlled experimental conditions designed to minimize measurement error.

Although the absolute discrepancy between the two estimates of energy balance is on the order of approximately 100 kcal/day, the corresponding relative errors of approximately 30–36% are large from the perspective of quantitative model validation (1–3). In quantitative modeling, discrepancies of this magnitude would typically raise concerns regarding the fidelity of the governing equations or the interpretation of the measurements used to evaluate them (52, 53). Importantly, the energy balance framework itself requires that the two estimates agree, because both are intended to quantify the same physical quantity, namely the net rate of change of stored body energy. Consequently, even when measurement uncertainty is acknowledged, discrepancies of this magnitude warrant closer examination of the assumptions underlying the model.

For this reason, the subsequent sections develop additional numerical consistency tests that are less sensitive to relatively modest absolute errors in energy estimation and instead examine directional predictions and macronutrient balance relationships implied by the EBM. These tests provide a more stringent evaluation of the quantitative implications of the model under controlled experimental conditions.

### 2.5 Results of the second numerical consistency test

**Figure 1:**
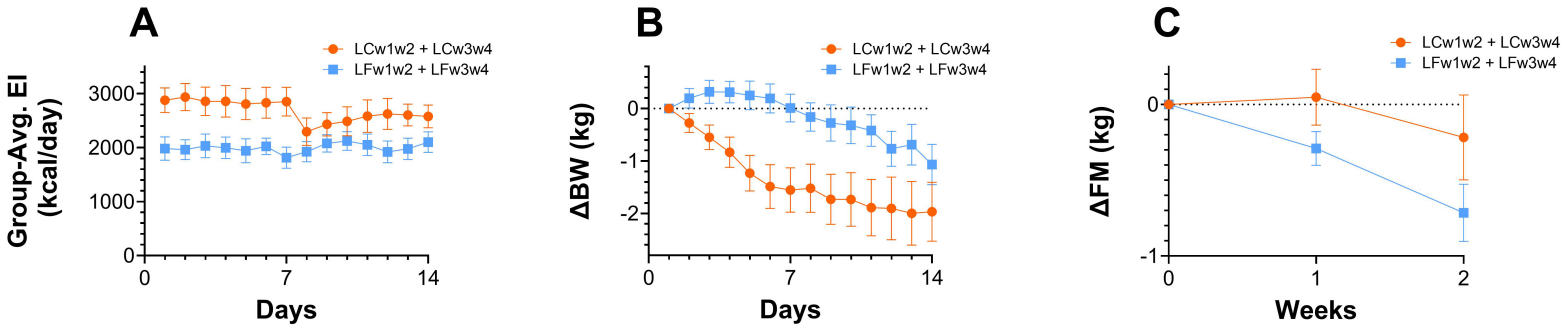
The numerical attributes of Hall et al.'s (39) open dataset are consistent with those in the original study. The open dataset of Hall et al. (39), available at Open Science Framework (https://osf.io/zdwqb/), only includes 16 subjects (9 males, 7 females) as 4 of the 20 participants dissent to share their information. If diet order is not considered, the obtained results are not different to those reported in the original publication. In all panels the orange color combines the low-carbohydrate diet (LC) of weeks 1 (w1) and 2 (w2) with that of weeks 3 (w3) and 4 (w4). The blue color combines the low-fat diet (LF) of w1 and w2 with that of w3 and w4. Values in all graphs are expressed as mean ± standard error (SE) as in the original report. **(A)** Group-average (avg.) daily *EI*. **(B)** Group-average body weight (*BW*) change. **(C)** Group-average *FM* change. Within-subject repeated DXA measurements of fat mass demonstrate that only the LF diet resulted in fat loss (one-tailed paired *t*-test: LC (*n* = 16): Δ*FM* = −0.217 ± 1.12 kg, *P* = 0.226; one-tailed paired *t*-test: LF (*n* = 16): Δ*FM* = −0.715 ± 0.76 kg, *P* = 0.000893).

Hall et al. (39) conducted an inpatient metabolic ward study in 20 non-diabetic adults (11 males, 9 females) to examine how the relative proportions of dietary carbohydrate and fat influence appetite control during unrestricted food consumption. Participants were randomly assigned to one of two treatment sequences: 14 days on a plant-based low-fat diet (LF) followed immediately, with no washout period, by 14 days on an animal-based ketogenic low-carbohydrate diet (LC), or the reverse sequence, LC followed by LF with no washout period. The authors reported no statistically significant effects of diet order (*P* = 0.32) or gender (*P* = 0.13), and therefore combined the data from both phases in their primary analysis (Figure 1). An important limitation of this design is the absence of a washout period between dietary phases. Consequently, physiological adaptations induced by the first diet may have persisted into the second phase, potentially influencing the metabolic and body composition responses observed after crossover. Such carryover effects could contribute to variability in the quantitative estimates obtained during the final phase of the trial and should therefore be considered when interpreting those results. However, this limitation does not affect the initial phase analyses, which were obtained before the crossover occurred.

In contrast, the analysis below considers the two phases separately: the initial phase from day 1 to day 14 and the final phase from day 15 to day 28. This separation allows evaluation of whether the EBM satisfies a second numerical consistency requirement under controlled conditions. This test is derived from an alternative form of the EBM governing equation (Equation 3), which predicts that individuals with similar body mass energy densities and comparable energy imbalances should exhibit similar changes in body mass. Unlike the first numerical test, which evaluates agreement between independent estimates of energy balance, the present test examines whether the model correctly predicts both the direction and magnitude of body mass change. The analysis therefore provides a direct empirical assessment of the internal coherence of the EBM framework when applied to high-quality metabolic-ward data.

**Figure 2:**
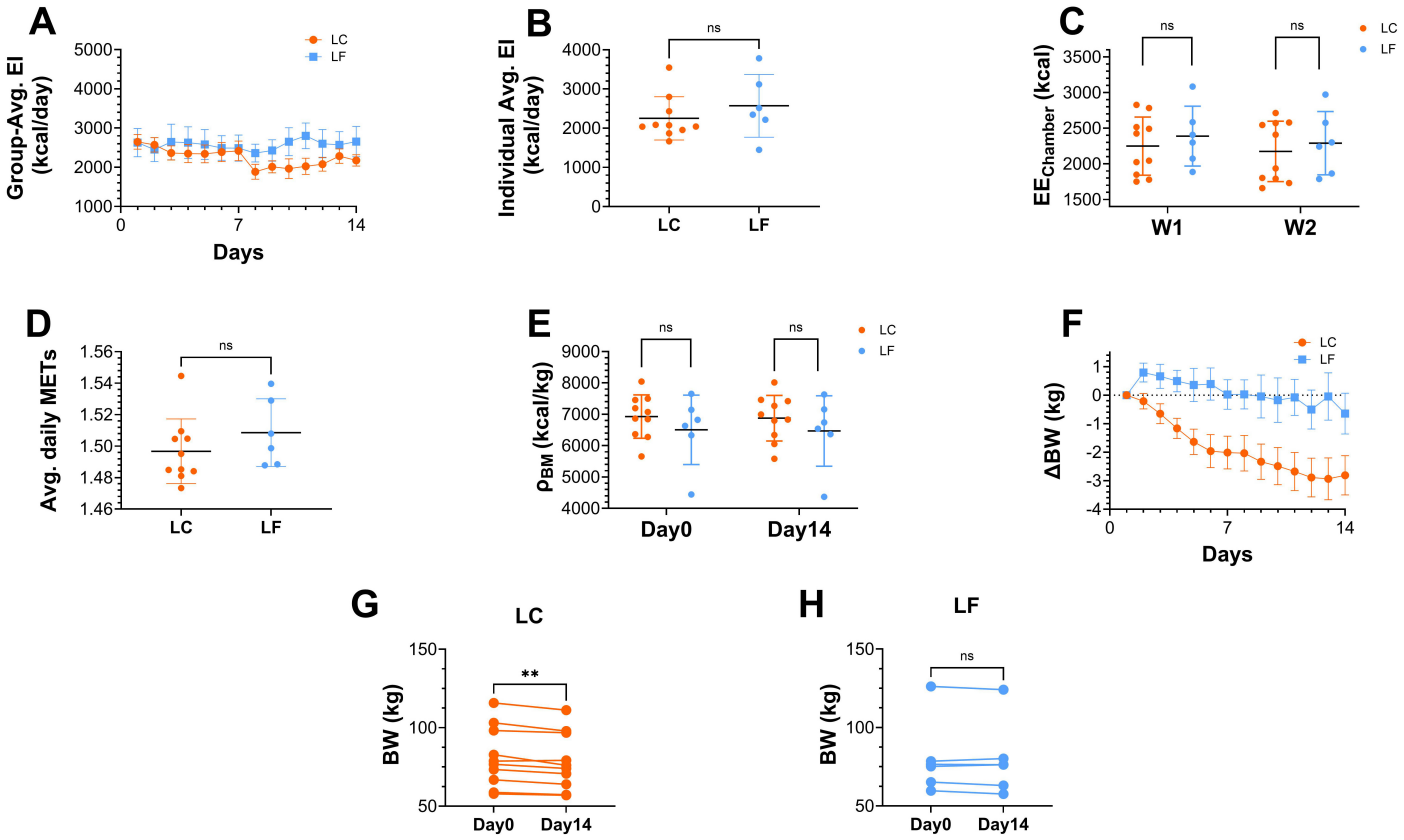
Initial phase data from Hall et al.'s (39) randomized controlled trial. **(A)** Daily group-average *EI*. Error bars: SE. **(B)** Average daily *EI* for individual participants during the initial phase of the trial. LC (*n* = 10): 2,250 ± 550 kcal/day vs. LF (*n* = 6): 2,571 ± 801 kcal/day, two-tailed unpaired *t*-test, ns: non-significant, *P* = 0.357. **(C)** Trial participants were confined to a metabolic chamber once per week (w) to measure their daily *EE*. W1: LC (*n* = 10): 2,249 ± 408 kcal/day vs. LF (*n* = 6): 2,389 ± 419 kcal/day, two-tailed unpaired *t*-test, *P* = 0.521. W2: LC (*n* = 10): 2,174 ± 424 kcal/day vs. LF (*n* = 6): 2,291 ± 443 kcal/day, two-tailed unpaired *t*-test, *P* = 0.609. **(D)** Physical activity expressed as average daily metabolic equivalents (METs, measured by accelerometry). LC (*n* = 10): 1.497 ± 0.0206 vs. LF (*n* = 6): 1.509 ± 0.0216, two-tailed unpaired *t*-test, *P* = 0.291. **(E)** Body mass energy density (ρ_*BM*_) at days 0 and 14. Here ρ_*FM*_ = 9,300 kcal/kg (38), ρ_*FFM*_ = 1,100 kcal/kg (38), and *dFFM/dFM* =10.4/*FM* (48). Day 0: LC (*n* = 10): 6,927 ± 692 kcal/kg vs. LF (*n* = 6): 6,505 ± 1,107 kcal/kg, two-tailed unpaired *t*-test, *P* = 0.359; Day 14: LC (*n* = 10): 6,876 ± 727 kcal/kg vs. LF (*n* = 6): 6,467 ± 1,124 kcal/kg, two-tailed unpaired *t*-test, *P* = 0.388. **(F)** Average change in *BW*. Error bars: SE. **(G)** The LC diet (*n* = 10) resulted in a significant mean weight change of −2.809 ± 2.18 kg. One-tailed paired *t*-test, **P = 0.00139. **(H)** The LF diet (*n* = 6) resulted in a non-significant mean weight change of −0.6467 ± 1.75 kg. One-tailed paired *t*-test, *P* = 0.203.

Figure 2 presents the initial phase data used to perform numerical consistency test number 2. Panels 2A and 2B show that the differences in energy intake between the LC and LF diets during the first 2 weeks were not statistically significant. Panels 2C and 2D indicate that energy expenditure and physical activity measurements were also comparable between the two diet conditions. Moreover, the body mass energy density over the initial phase did not differ between the LC and LF groups and remained unaltered (Figure 2E). Consequently, all numerical inputs to the second form of the EBM governing equation (Equation 3) are of comparable magnitude for both diets, and the model therefore predicts similar changes in body mass for the two groups. Because the objective of the present numerical consistency test is to evaluate whether the EBM reproduces the mean response expected from the measured group-average energy imbalance, predictions derived from group-average measurements are compared with the corresponding group-average changes in body weight. Accordingly, the analysis evaluates the numerical consistency of the EBM at the group level rather than the variability of individual subject responses.

Under these conditions, Equation 3 can be directly integrated over the 14-day interval to obtain explicit predictions for the expected change in body weight:


$$\begin{array}{l} \text{Δ}BW=\frac{\left[\langle EI\rangle -\langle EE\rangle \right]\cdot T}{\rho_{BM}} \end{array}$$ (6)

Using the mean values in Figures 2B, C, E yield the following EBM's forecast:

LC prediction:

$\text{Δ}BW=\frac{\left[2,250\frac{\text{kcal}}{\text{day}}-\frac{1}{2}\left(2,249\frac{\text{kcal}}{\text{day}}+2,174\frac{\text{kcal}}{\text{day}}\right)\right]\cdot 14\text{ days}}{12\left(\text{6,927}\frac{\text{kcal}}{\text{kg}}\text{+6,876}\frac{\text{kcal}}{\text{kg}}\right)}\approx +0.078\text{kg}$

LF prediction:

$\text{Δ}BW=\frac{\left[2,571\frac{\text{kcal}}{\text{day}}-\frac{1}{2}\left(2,389\frac{\text{kcal}}{\text{day}}+2,291\frac{\text{kcal}}{\text{day}}\right)\right]\cdot 14\text{ days}}{12\left(\text{6,505}\frac{\text{kcal}}{\text{kg}}\text{+6,467}\frac{\text{kcal}}{\text{kg}}\right)}\approx +0.498\text{k}g$

The corresponding absolute and relative errors are:

LC:

*AE* = |0.078−(−2.809)| = 2.887kg;

$RE=|\frac{0.078-\left(-2.809\right)}{-2.809}|\times 100\approx 103\%$

LF:

*AE* = |0.498−(−0.6467)| = 1.1447kg;

$RE=|\frac{0.498-\left(-0.6467\right)}{-0.6467}|\times 100\approx 177\%$

As the reader may verify, applying the Basolo et al. (51) corrections for unabsorbed energy intake does not materially change the quantitative results. Consistent with these inputs, the EBM yields predictions of statistically indistinguishable weight changes for both dietary groups, corresponding to a small weight gain over the 14-day interval given the small magnitude of the measured positive energy imbalances. In contrast, the empirical observations differ from these predictions in both direction and magnitude. The LC group experienced a reduction of approximately 2.809 kg in total body mass during the initial phase (Figures 2F, G), whereas the LF group's body mass remained stable (Figures 2F, H). This divergence between predicted and observed outcomes indicates that the quantitative predictions of the EBM are not consistently supported by the experimental data.

A potential explanation for this discrepancy is that short-term dietary interventions may involve physiological adaptations such as glycogen depletion, associated water shifts, and transient metabolic adjustments (19, 42, 43). Such factors can influence short-term body weight measurements and must therefore be considered when interpreting the results. These effects are implicitly accounted for in Equation 3 through changes in body mass energy density (ρ_*BM*_), which allows individuals with different body composition dynamics to exhibit different magnitudes of weight change. However, in the present case, ρ_*BM*_ values were similar between the two dietary groups, indicating that such effects cannot account for the observed divergence. Furthermore, as discussed in the following section, the reduction observed in the LC group also included a measurable decrease in fat mass, indicating that the observed weight change cannot be explained solely by glycogen depletion or associated water shifts.

**Figure 3:**
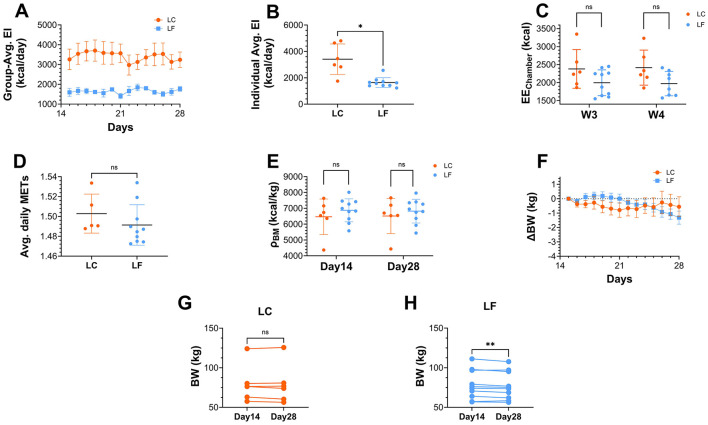
Final phase data from Hall et al.'s (39) randomized controlled trial. **(A)** Daily group-average *EI*. Error bars: SE. **(B)** Average daily *EI* for individual participants during the final phase of the trial. LC (*n* = 6): 3,414 ± 1,161 kcal/day vs. LF (*n* = 10): 1,645 ± 370 kcal/day, two-tailed unpaired *t*-test, *P = 0.0124. **(C)** Trial participants were confined to a metabolic chamber once per week (w) to measure their daily *EE*. W3: LC (*n* = 6): 2,380 ± 536 kcal/day vs. LF (*n* = 10): 1,996 ± 357 kcal/day, two-tailed unpaired *t*-test, ns: non-significant, *P* = 0.107. W4: LC (*n* = 6): 2,416 ± 487 kcal/day vs. LF (*n* = 10): 1,972 ± 379 kcal/day, two-tailed unpaired *t*-test, *P* = 0.0666. **(D)** Physical activity expressed as average daily metabolic equivalents (METs, measured by accelerometry). LC (*n* = 6): 1.503 ± 0.0216 vs. LF (*n* = 10): 1.491 ± 0.0202, two-tailed unpaired *t*-test, *P* = 0.291. **(E)** Body mass energy density (ρ_*BM*_) at days 14 and 28. Here ρ_*FM*_ = 9,300 kcal/kg (38), ρ_*FFM*_ = 1,100 kcal/kg (38), and *dFFM/dFM* =10.4/*FM* (48). Day 14: LC (*n* = 6): 6,467 ± 1,124 kcal/kg vs. LF (*n* = 10): 6,876 ± 727 kcal/kg, two-tailed unpaired *t*-test, *P* = 0.388; Day 28: LC (*n* = 6): 6,515 ± 1,110 kcal/kg vs. LF (*n* = 10): 6,823 ± 749 kcal/kg, two-tailed unpaired *t*-test, *P* = 0.516. **(F)** Average change in *BW*. Error bars: SE. **(G)** The LC diet (*n* = 6) resulted in a non-significant mean weight change of −0.5617 ± 1.7 kg. One-tailed paired *t*-test, *P* = 0.227. **(H)** The LF diet (*n* = 10) resulted in a significant mean weight change of −1.318 ± 1.44 kg. One-tailed paired *t*-test, **P = 0.0089.

Figure 3 illustrates the final phase of the trial. Panels 3A, 3B, and 3C show that the LC diet produced a substantial positive energy balance (~ +1,000 kcal/day), whereas the LF diet resulted in a negative energy balance (~ −339 kcal/day). Physical activity and body mass energy density were again comparable between the two diets (Figures 3D, E). According to the second version of the EBM governing equation (Equation 3), the LC diet should therefore lead to weight gain, whereas the LF diet should produce weight loss. Using the mean values in Figures 3B, C, E yields the following forecasts:

LC prediction:

$\text{Δ}BW=\frac{\left[0.93\times 3,414\frac{kcal}{day}-\frac{1}{2}\left(2,380\frac{kcal}{day}+2,416\frac{kcal}{day}\right)\right]\cdot 14days}{\frac{1}{2}\left(\text{6,466}\text{.9}\frac{kcal}{kg}\text{+6,514}\text{.6}\frac{kcal}{kg}\right)}\approx +1.68kg$

LF prediction:

$\text{Δ}BW=\frac{\left[0.89\times 1,645\frac{kcal}{day}-\frac{1}{2}\left(1,996\frac{kcal}{day}+1,972\frac{kcal}{day}\right)\right]\cdot 14days}{\frac{1}{2}\left(\text{6,876}\text{.4}\frac{kcal}{kg}\text{+6,822}\text{.6}\frac{kcal}{kg}\right)}\approx -1.06kg$

The resulting absolute and relative errors are:

LC:

*AE* = |1.68−(−0.5617)| = 2.2417kg;

$RE=|\frac{1.68-\left(-0.5617\right)}{-0.5617}|\times 100\approx 399\%$

LF:

*AE* = |−1.06−(−1.318)| = 0.258kg;

$RE=|\frac{-1.06-\left(-1.318\right)}{-1.318}|\times 100\approx 20\%$

In this case, omitting the Basolo et al. (51) corrections for unabsorbed energy intake results in even larger discrepancies.

As in the initial phase of the trial, the predicted outcomes differ from the empirical observations. Participants assigned to the LC diet remained weight stable (Figures 3F, G), whereas the predicted weight loss for the LF diet underestimated the measured change by approximately 20% (Figures 3F, H). Taken together, the results shown in Figures 2, 3 indicate that the direction and magnitude predictions derived from Equation 3 do not quantitatively match the empirical outcomes reported in Hall et al. (39), although the final phase results should be interpreted in light of the potential carryover effects inherent to the crossover design. These findings therefore suggest limitations in the ability of the energy balance framework to reproduce the observed body weight dynamics under the experimental conditions examined.

To evaluate whether uncertainty in the measured quantities could account for the discrepancies observed in the second numerical consistency test, a sensitivity analysis was performed (Supplementary Table S1). The analysis quantified the magnitude of correction required in the measured energy balance to reconcile the EBM predictions with the observed body weight changes. For the LF final phase, where the predicted and observed weight changes shared the same direction, the discrepancy could be reduced by relatively modest adjustments within the range of plausible measurement uncertainty. In contrast, the LC initial and LC final phases required substantially larger corrections. In the LC initial phase, the EBM prediction of slight weight gain was opposite to the observed weight loss, implying that the measured energy balance would need to shift from a near-neutral balance (+38.5 kcal/day) to a large negative balance (approximately −1,385 kcal/day) before agreement could be obtained. Similarly, reconciliation of the LC final phase required the measured energy balance to shift from a substantial positive balance (+777 kcal/day) to a negative balance (approximately −261 kcal/day). In comparison, the LF final phase required a correction of only approximately −125 kcal/day to reproduce the observed weight change. These findings indicate that, although measurement uncertainty may influence the quantitative magnitude of some discrepancies, reconciliation of the LC-phase results requires substantial revisions to the underlying measurements and is therefore not readily explained by ordinary measurement uncertainty alone.

### 2.6 Results of the third numerical consistency test

The third numerical consistency test evaluates whether the observed changes in fat mass correspond to the fat balance as defined by the EBM (Equation 4) and as typically applied in controlled metabolic studies (49). In the EBM framework, changes in fat mass are determined by the difference between fat intake and net fat oxidation. When this relationship is expressed over a finite time interval, the cumulative change in fat mass equals the product of the average daily fat balance and the duration of the intervention (Equation 5). This relationship provides a direct test of whether the macronutrient fluxes reported in the experimental dataset are consistent with the observed changes in body composition. Specifically, if the average daily fat balance is positive over a given interval, the model predicts an increase in fat mass over that interval; conversely, a negative fat balance should produce a decrease in fat mass.

**Figure 4:**
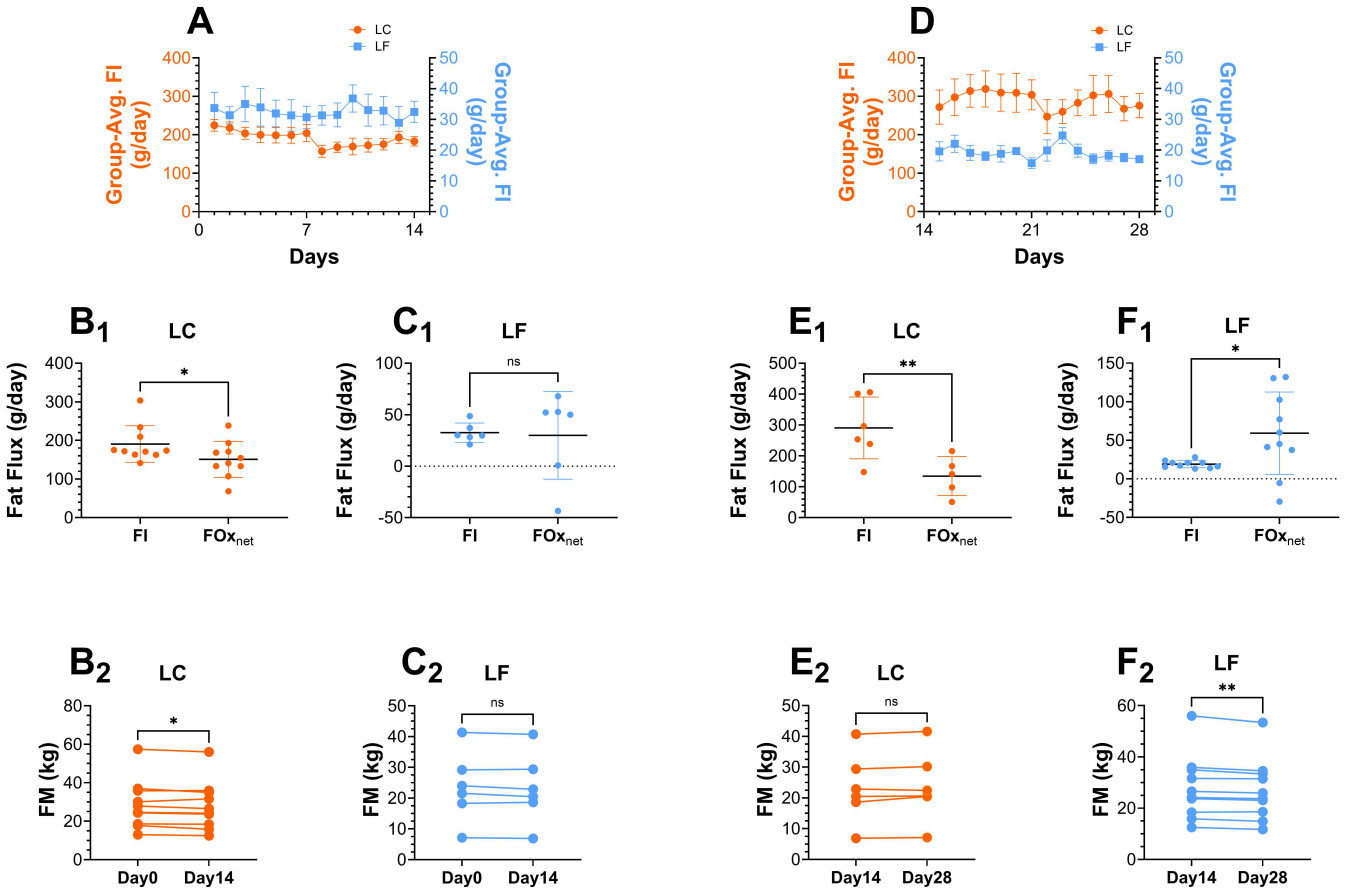
Fat mass dynamics from Hall et al.'s (39) randomized controlled trial. **(A)** Initial phase average fat intake (*FI*). Error bars: SE. **(B**_**1**_**)** LC diet fat balance. Each dot in the *FI* column represents the average *FI* of an individual participant during the initial phase of the trial. Dots in the *FOx*_*net*_ column represent the corresponding average net fat oxidation, calculated from the two metabolic chamber visits conducted during the same period. *FI* (*n* = 10): 191 ± 47 g/day vs. *FOx*_*net*_ (*n* = 10): 151 ± 47 g/day, one-tailed unpaired *t*-test, *P = 0.0376. **(B**_**2**_**)** The LC diet (*n* = 10) resulted in a significant mean change in *FM* of −0.688 ± 1.04 kg, as assessed by DXA. One-tailed paired *t*-test, *P = 0.0339. **(C**_**1**_**)** LF diet fat balance. Panel details are as in B_1_. A negative *FOx*_*net*_ indicates fat synthesis, as net oxidation is the difference between substrate oxidation and synthesis (50). *FI* (*n* = 6): 32.5 ± 9.8 g/day vs. *FOx*_*net*_ (*n* = 6): 30 ± 42 g/day, one-tailed unpaired *t*-test, ns: non-significant, *P* = 0.446. **(C**_**2**_**)**. The LF diet (*n* = 6) resulted in a non-significant mean change in *FM* of −0.424 ± 1.49 kg, as assessed by DXA. One-tailed paired *t*-test, *P* = 0.0777. **(D)** Final phase average *FI*. Error bars: SE. LC: *n* = 6; LF: *n* = 10. **(E**_**1**_**)** LC diet fat balance. Panel details are as in B_1_. *FI* (*n* = 6): 291 ± 100 g/day vs. *FOx*_*net*_ (*n* = 5) 134 ± 63 g/day, one-tailed unpaired *t*-test, **P = 0.0073. **(E**_**2**_**)** The LC diet (*n* = 6) resulted in a non-significant mean change in *FM* of +0.562 ± 0.78 kg, as assessed by DXA. One-tailed paired *t*-test, *P* = 0.068. **(F**_**1**_**)** LF diet fat balance. Panel details are as in B_1_. *FI* (*n* = 10): 19 ± 4.74 g/day vs. *FOx*_*net*_ (*n* = 10): 59 ± 54 g/day, one-tailed unpaired *t*-test, *P = 0.0147. **(F**_**2**_**)** The LF diet (*n* = 10) resulted in a significant mean change in *FM* of −0.89 ± 0.79 kg, as assessed by DXA. One-tailed paired *t*-test, **P = 0.0033.

Figure 4A shows the time course of the group-average daily fat intake for both diet groups during the initial phase of the Hall et al. (39) trial. In the LC diet, the mean fat intake was approximately 191 g/day, whereas the mean net fat oxidation was approximately 151 g/day (Figure 4B_1_). Under these conditions, the implied average daily fat balance is positive. Over the 14-day intervention interval, this balance corresponds to a predicted increase in fat mass of approximately


$$\begin{array}{l} \text{Δ}FM=\left[\langle FI\rangle -\langle FOx_{net}\rangle \right]\cdot T\\ \;=\left[191\frac{\text{g}}{\text{day}}-151\frac{\text{g}}{\text{day}}\right]\times 14\text{ days = +560g = +0 .56kg .} \end{array}$$

However, the empirical observations differ from this prediction. Participants assigned to the LC diet did not gain fat mass during the initial phase; instead, the group experienced an average fat mass reduction of approximately 0.688 kg (Figure 4B_2_).

In contrast, participants assigned to the low-fat (LF) diet consumed an average of approximately 32.5 g/day of dietary fat while oxidizing approximately 30 g/day (Figure 4C_1_). Under these conditions, the predicted fat balance is close to zero


$$\begin{array}{l} \text{Δ}FM=\left[32.5\frac{\text{g}}{\text{day}}-30\frac{\text{g}}{\text{day}}\right]\times 14\text{ days}=\text{+35g = +0 .035kg}, \end{array}$$

implying little or no change in fat mass over the intervention interval. Consistent with this prediction, the measured fat mass of the LF group remained stable during the initial phase (Figure 4C_2_).

Figure 4D presents the corresponding analysis for the final phase of the trial. During this phase, the LC diet produced a substantially higher fat intake, with an average of approximately 291 g/day, while the reported net fat oxidation averaged approximately 134 g/day (Figure 4E_1_). This difference implies a strongly positive fat balance during the final phase of the intervention. Over the 14-day interval, the predicted cumulative fat gain is approximately


$$\begin{array}{l} \text{Δ}FM=\left[291\frac{\text{g}}{\text{day}}-134\frac{\text{g}}{\text{day}}\right]\times 14\text{ days = +}2,198\text{g = +}2.198\text{kg .} \end{array}$$

Nevertheless, the measured fat mass of the LC group remained stable during this phase (Figure 4E_2_).

In the LF diet, the average fat intake was 19 g/day, while the average net fat oxidation was 59 g/day (Figure 4F_1_). Therefore, during the final phase, the LF group's fat mass is expected to decrease by approximately


$$\begin{array}{l} \text{Δ}FM=\left[19\frac{\text{g}}{\text{day}}-59\frac{\text{g}}{\text{day}}\right]\times 14\text{ days =}-\text{560g =}-\text{0 .56kg .} \end{array}$$

Participants assigned to the LF diet did experience a decrease in fat mass during this phase; however, the observed reduction was approximately 59% larger than the predicted value (Figure 4F_2_).

It is important to note that short-term dietary interventions may involve physiological processes such as glycogen depletion, associated water shifts, and transient metabolic adjustments that can influence body weight measurements (19, 42, 43). However, such mechanisms primarily affect glycogen stores and body water rather than adipose tissue mass. Because the analysis in this section focuses specifically on measured changes in fat mass, the discrepancies observed between predicted fat balance and measured fat mass dynamics cannot be explained solely by short-term shifts in glycogen or body water.

Collectively, the comparisons shown in Figure 4 indicate that the fat balance relationships implied by the EBM are not consistently reproduced by the observed fat mass changes. In several instances, the model fails not only to predict the magnitude of fat mass change but also its direction, with positive predicted fat balance coinciding with fat loss and strongly positive predicted balance failing to produce the expected fat gain. Even when the direction of change is correctly predicted, the magnitude of the response deviates substantially from the measured outcome. These findings therefore indicate a lack of both quantitative and directional agreement between the EBM's fat balance predictions and the observed body composition changes under the experimental conditions examined.

To evaluate whether glycogen-associated water shifts, DXA uncertainty, or errors in indirect-calorimetry-derived estimates of substrate oxidation could account for the discrepancies observed in the third numerical consistency test, a sensitivity analysis was performed (Supplementary Table S2). The analysis quantified the magnitude of measurement bias required to reconcile the EBM fat balance predictions with the observed changes in fat mass. For the LF phases, the discrepancies were generally smaller and could be reduced substantially under plausible assumptions regarding measurement uncertainty. In contrast, the LC phases exhibited larger discrepancies, including a directional disagreement during the initial phase and a substantial magnitude disagreement during the final phase.

During the LC initial phase, the EBM predicted a fat mass gain of approximately 0.56 kg, whereas the measured change indicated a fat mass loss of approximately 0.688 kg. Eliminating this directional disagreement required either a DXA correction approaching 0.7 kg or a substantial increase in the estimated rate of fat oxidation. Even after allowing a favorable DXA correction of 0.5 kg, the estimated rate of fat oxidation would still need to be approximately 35% higher than the reported value to reconcile the prediction with the observed outcome. During the LC final phase, the EBM correctly predicted the direction of change but substantially overestimated its magnitude, forecasting a fat mass gain of approximately 2.20 kg compared with an observed gain of approximately 0.56 kg. Reconciling these values required increases in the estimated rate of fat oxidation ranging from approximately 34% to 87%, depending on the assumed level of DXA uncertainty.

Taken together, the sensitivity analysis indicates that measurement uncertainty may partially reduce some of the quantitative discrepancies observed in the LF phases. However, the principal discrepancies observed in the LC phases persist unless substantial bias is assumed in the underlying measurements. These findings therefore suggest that the inconsistencies identified in the third numerical consistency test cannot be readily attributed to the levels of uncertainty typically associated with glycogen-associated water shifts, DXA-derived body composition measurements, or indirect-calorimetry-derived estimates of substrate oxidation.

### 2.7 Weak empirical correspondence between energy imbalance and weight change

The results of the three numerical consistency tests presented above reveal a consistent pattern. Across independent estimates of energy balance, body mass dynamics, and fat mass changes, the quantitative correspondence between EBM predictions and observed outcomes is not consistently reproduced under tightly controlled experimental conditions. Rather than displaying the precise and reproducible relationships expected of a quantitative predictive framework, the empirical results exhibit substantial variability in both the direction and magnitude of the association between measured energy imbalance and observed body mass change.

**Figure 5:**
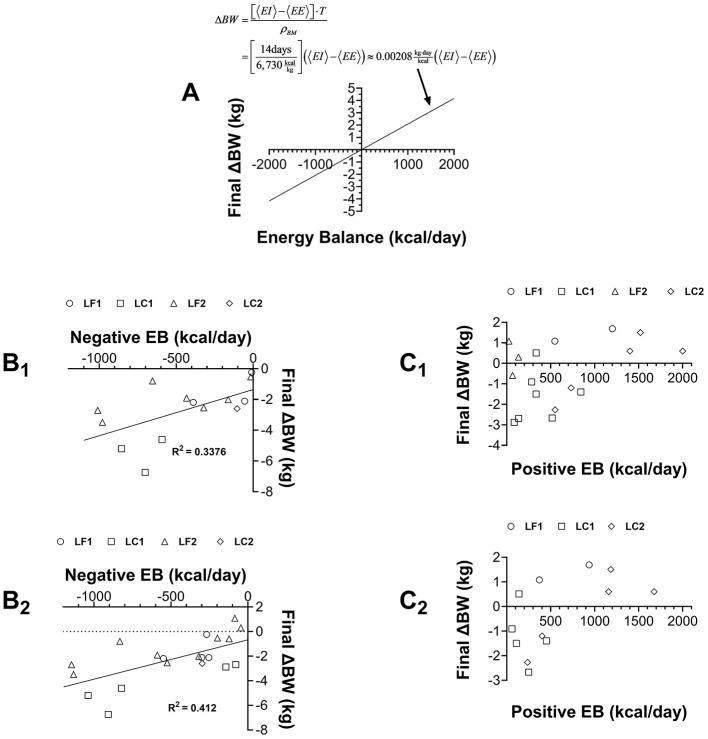
Individual-level relationship between energy imbalance and weight change. **(A)** Integration of the second version of the EBM governing equation (Equation 3) over a period *T*, assuming constant body mass energy density (ρ_*BM*_), yields Equation 6 (shown equation). The Hall et al. (39) dataset satisfies these assumptions, allowing evaluation of the predicted relationship between energy imbalance (kcal/day) and weight change. In this analysis, body mass energy density is taken as the average value across the study (~6,730 kcal/kg), as no significant differences were observed between trial phases (Figures 2E, 3E). **(B**_**1**_**)** In the open dataset from Hall et al. (39), all negative energy balances (uncorrected for unabsorbed energy intake) are associated with weight loss. The scatter plot and corresponding coefficient of determination (*R*^2^) describe the relationship between energy imbalance and weight change. The solid line represents the least-squares regression fit (slope: 0.003 kg·day/kcal; intercept: −1.3576 kg). EB: energy balance. **(B**_**2**_**)** After applying corrections for unabsorbed energy intake (7% reduction for weight gain; 11% for weight loss), the adjusted data from B_1_ are shown. The regression line has a slope of 0.0032 kg·day/kcal and an intercept of −0.6719 kg. **(C**_**1**_**)** In the same dataset, several observations (9 of 17; ~53%) show weight loss occurring during periods of positive energy balance (uncorrected). **(C**_**2**_**)** After applying corrections for unabsorbed energy intake, the number of observations with positive energy balance is reduced; however, weight loss during positive energy balance remains present in a subset of participants (6 of 12). LF1, initial phase LF participant; LC1, initial phase LC participant; LF2, final phase LF participant; LC2, final phase LF participant.

Figure 5 illustrates the relationship between energy imbalance and observed weight change for individual participant intervals in the Hall et al. (39) dataset. Although Equation 6 predicts a strong linear relationship between these quantities (Figure 5A), the empirical association between negative energy balance and the magnitude of weight loss appears weak and highly variable (Figures 5B_1_, B_2_). These results indicate that the magnitude of weight change cannot be reliably inferred from the measured level of energy imbalance, even under tightly controlled experimental conditions.

An additional feature of the dataset concerns the direction of weight change under conditions of positive energy balance. As shown in Figures 5C_1_, C_2_, half of the intervals characterized by positive energy balance are nevertheless associated with reductions in body weight over the corresponding 14-day period, ranging from approximately 0.9 kg to 2.7 kg. Under the conventional interpretation of the EBM, sustained positive energy balance would be expected to produce weight gain rather than weight loss. The occurrence of weight reduction under these conditions therefore represents a directional inconsistency between the model's predictions and the observed outcomes.

Taken together, these observations indicate that the empirical relationship between energy imbalance and body mass change is both weaker and more variable than expected from a quantitatively predictive model. The fact that these discrepancies arise under tightly controlled experimental conditions further suggests limitations in the ability of energy imbalance alone to account for short-term body weight dynamics. More broadly, because quantitative predictive frameworks are expected to reproduce controlled experimental observations, such discrepancies raise questions regarding the predictive reliability of the model.

### 2.8 Systematic relationships between mass intake, weight change, diet order, and diet composition

The preceding analyses of the trial by Hall et al. (39) indicate that variations in energy imbalance do not consistently account for the observed changes in body weight and fat mass under controlled experimental conditions. This raises the question of whether alternative measurable quantities within the same dataset exhibit a more systematic correspondence with body mass dynamics. In this context, food mass intake represents a directly measured physical quantity that is distinct from energy intake. If body mass change reflects the balance between material inflow and outflow, then variation in total mass intake would be expected to align more closely with observed changes in body weight.

**Figure 6:**
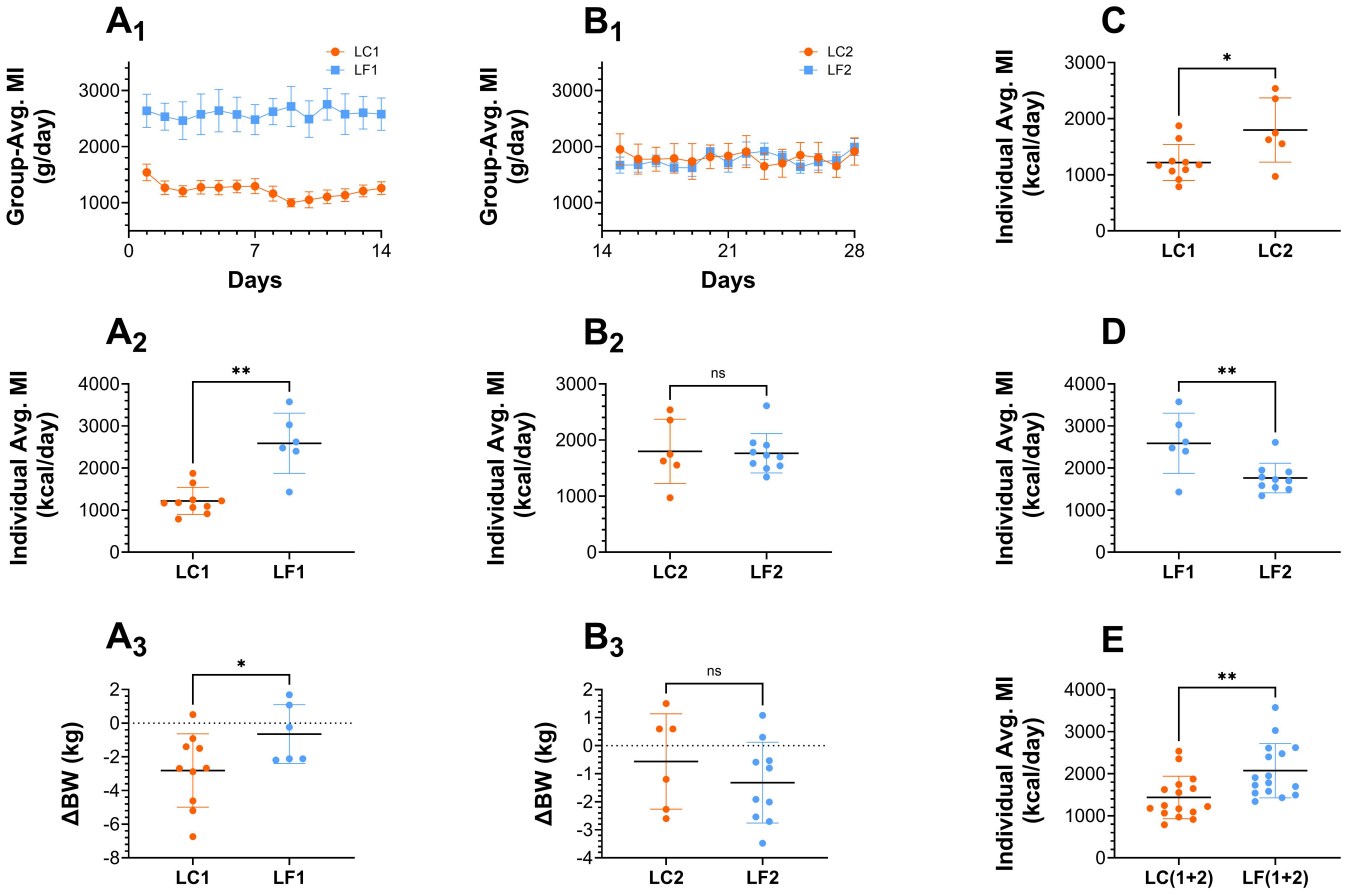
Relationships between food mass intake, weight change, diet order, and diet composition. **(A**_**1**_**)** Initial phase [1] daily group-average water-free food mass intake (*MI*). Error bars: SE. **(A**_**2**_**)** Average *MI* for each participant during the initial phase of the trial. LC1 (*n* = 10): 1,220 ± 322 g/day vs. LF1 (*n* = 10): 2,589 ± 715 g/day, two-tailed unpaired *t*-test, **P = 0.004. **(A**_**3**_**)** Final weight change contrast for the initial phase. LC1 (*n* = 10): −2.809 ± 2.18 kg vs. LF1 (*n* = 6): – 0.6467± 1.75 kg, one-tailed unpaired *t*-test, *P = 0.0294. **(B**_**1**_**)** Final phase [2] daily group-average *MI*. Error bars: SE. **(B**_**2**_**)** Average *MI* for each participant during the final phase of the trial. LC2 (*n* = 6): 1,798 ± 572 g/day vs. LF2 (*n* = 10): 1,764 ± 352 g/day, one-tailed unpaired *t*-test, ns: non-significant, *P* = 0.179. **(B**_**3**_**)** Final weight change contrast for the final phase. LC2 (*n* = 6): −0.5617 ± 1.7 kg vs. LF2 (*n* = 10): −1.318 ± 1.44 kg, one-tailed unpaired *t*-test, ns: non-significant, *P* = 0.357. **(C)** MI contrast between initial and final LC diets. LC1 vs. LC2, two-tailed unpaired *t*-test, *P = 0.0204. **(D)** MI contrast between initial and final LF diets. LF1 vs. LF2, two-tailed unpaired *t*-test, **P = 0.00749. **(E)** MI contrast between combined (initial + final: 1+2) LC and combined LF. LC(1+2) (*n* = 16): 1,497 ± 505 g/day vs. LF(1+2) (*n* = 16): 2,074 ± 644 g/day, two-tailed unpaired *t*-test, **P = 0.00404.

This possibility can be examined directly using the experimental data. During the initial phase of the intervention, energy intake was statistically similar between the LF and LC diets (Figures 2A, B). Despite this similarity, the LC diet was associated with substantial reductions in body weight and fat mass (Figures 2F, G, 4B_2_), whereas participants assigned to the LF diet remained weight stable and exhibited no significant change in fat mass (Figures 2F, H, 4C_2_). In contrast, food mass intake differed markedly between the diets. Participants consuming the LC diet ingested substantially less total food mass than those consuming the LF diet (Figures 6A_1_, A_2_). Consequently, variation in food mass intake aligned more closely with the observed differences in body composition than did variation in food energy intake (Figures 6A_1_-A_3_).

A similar pattern emerges during the final phase of the trial. In this phase, energy intake differed substantially between the two diets (Figures 3A, B). However, total food mass intake was comparable between treatments, and the resulting weight changes were correspondingly similar across diets (Figures 6B_1_-B_3_). Thus, large differences in energy intake were not accompanied by corresponding differences in body weight when total mass intake remained comparable. These observations are consistent with expectations derived from mass balance considerations (40, 41). When mass elimination is comparable between dietary conditions, the diet with lower total mass intake is associated with greater weight reduction (Figure 6A_3_). Conversely, when both mass intake and mass elimination are approximately equal between treatments, whether at zero or non-zero balance, the resulting weight changes are expected to be statistically indistinguishable (Figure 6B_3_).

Diet order further reinforces this pattern. When an LC diet followed an LF diet, it was associated with significantly higher food mass intake relative to the initial LC phase (Figure 6C). Conversely, when an LF diet followed an LC diet, it was associated with substantially lower mass intake relative to the initial LF phase (Figure 6D). Across both phases, however, the LC diet was associated with a significantly lower total unrestricted mass intake than the LF diet, indicating a systematic reduction in mass intake under LC feeding despite the order-dependent differences observed between phases (Figure 6E). These order effects are consistent with physiological and behavioral adaptations occurring during the initial dietary exposure, which may influence subsequent feeding behavior and thereby modulate total mass intake independently of the energy density of the food.

Taken together, these results reveal three consistent empirical patterns. First, variation in food mass intake shows a stronger and more systematic correspondence with observed changes in body weight than variation in food energy intake. Second, diet composition appears to influence total mass intake in ways that are not fully captured by differences in energy intake alone. Third, this pattern is particularly evident under unrestricted feeding conditions, where changes in body weight align more closely with variations in the quantity of matter ingested than with variations in its caloric content.

These observations suggest that food mass intake may provide a more direct measurable correlate of the processes governing body mass dynamics in this experimental context. Because body weight reflects the total quantity of matter contained within the system, quantities expressed in units of mass offer a physically transparent basis for interpreting changes in body weight. Within this framework, food mass intake can be viewed as a behavioral expression of appetite that directly determines the material balance governing body mass change.

### 2.9 Internal consistency analysis of the energy balance framework

Central to the EBM is the assertion that changes in body weight should exhibit a strong positive relationship with energy imbalance , as formalized in the second version of its governing equation (Equation 3). However, the empirical analyses presented in the preceding sections indicate that the quantitative correspondence between measured energy imbalance and observed weight change is weaker than expected, even under tightly controlled metabolic-ward conditions. These findings motivate a deeper question that extends beyond empirical prediction: does the commonly assumed equivalence between body weight stability and energy balance (*EI* = *EE*) remain internally consistent when the macronutrient fluxes underlying energy expenditure are represented explicitly? In practice, the EBM treats these two conditions as equivalent. By contrast, in thermodynamic analyses of open systems, energy balance and mass balance are treated as distinct state relationships governed by different conservation principles (54, 55). This distinction raises the possibility that treating these two conditions as equivalent within the EBM framework may introduce internal inconsistencies when macronutrient fluxes contributing to energy expenditure are considered explicitly.

Arencibia-Albite (41) previously examined this issue mathematically and showed that treating body mass balance and energy balance as equivalent conditions can generate internal inconsistencies within the EBM accounting framework. A concise updated formulation of that derivation is presented here. Notably, the conclusion follows directly from the accounting relationships of the EBM and does not depend on any particular dataset.

Because metabolic fluxes vary during feeding and fasting cycles, body weight stability is understood here in the long-term sense; that is, the average rate of body weight change over the interval considered is zero. Within the EBM, this condition is routinely interpreted as corresponding to energy balance (*EI* = *EE*) over the same interval (8–24). The following derivation examines whether that standard coincidence remains internally consistent when macronutrient fluxes are represented explicitly. In particular, the quantitative analysis below shows that when the coincidence of weight stability and energy balance is imposed, the EBM implies that absorbed dietary protein must be entirely utilized for energy production through oxidation or gluconeogenesis. Under these conditions, no portion of the absorbed protein remains available to replace the unavoidable loss of body protein associated with normal physiological turnover. The resulting state implies a condition in which body mass is mathematically stable according to the EBM accounting relationships while simultaneously decreasing through uncompensated protein loss, thereby generating an internally inconsistent outcome.

To demonstrate this result formally, the proposition under consideration and its interpretation are first stated, followed by the mathematical proof.

Proposition: *Within the accounting framework of the EBM, imposing the coincidence of body weight stability and energy balance (EI* = *EE) implies a persistent negative protein balance once obligatory protein loss is included*.

Interpretation: *Because a persistent negative protein balance implies ongoing loss of body mass in the form of protein, this result is incompatible with true body weight stability. Importantly, the proposition is not intended as a description of actual human physiology. Rather, it examines the consequences of imposing the standard EBM assumption that energy balance and body weight stability are equivalent conditions. Accordingly, any contradiction that emerges from the derivation should be interpreted as a logical inconsistency within the accounting structure of the EBM itself rather than as evidence that humans in weight stability experience persistent negative protein balance*.

Proof: The proof begins by defining protein balance (*PB*, g/day).

Protein balance results from the difference between the daily absorbed protein intake (*PI*_*a*_, g/day) and the total daily protein loss, which can be decomposed into the sum of net protein oxidation (*POx*_*net*_ = protein oxidation + gluconeogenesis, g/day) (50) and obligatory protein loss (OPL, g/day):


$$\begin{array}{l} PB=PI_{a}-POx_{net}-OPL \end{array}$$

In this expression, only *PI*_*a*_ and *POx*_*net*_ contribute to daily energy balance (21, 49). Specifically, after scaling these terms by the energy density of protein, *PI*_*a*_ represents the portion of the daily absorbed energy intake derived from protein, while *POx*_*net*_ corresponds to the fraction of the daily expended energy generated through net protein oxidation. In contrast, *OPL* represents an unavoidable daily loss of body mass in protein form, unrelated to adenosine triphosphate synthesis, and therefore makes no contribution to the daily energy expenditure (21, 56–58).

A key physical requirement of protein balance during prolonged weight stability is that it must be zero. Any persistent deviation would lead to a contradiction. For example, if body weight remains stable but the mean protein balance is chronically positive, then body mass would be augmenting in the form of accumulating protein, which implies increasing body weight and stability at the same time. Conversely, if body weight is stable while the mean protein balance is chronically negative, then body mass would be decreasing due to the loss of protein despite being assumed stable. Thus, weight stability logically requires that the long-term protein balance equals zero.

Next, note that when prolonged weight stability and energy balance occur simultaneously, the following accounting relations must both hold (21):

Weight Stability: mass inflow (g/day) = mass outflow (g/day)

Energy Balance: absorbed energy intake (kcal/day) = energy expenditure (kcal/day)

In mathematical form, these can be expressed as:


$$\begin{array}{l} \;FI_{a}+CI_{a}+PI_{a}=FOx_{net}+COx_{net}+POx_{net}+OPL\\ \rho_{F}FI_{a}+\rho_{C}CI_{a}+\rho_{P}PI_{a}=\rho_{F}FOx_{net}+\rho_{C}COx_{net}+\rho_{P}POx_{net}\;\left(s_{1}\right) \end{array}$$

where

ρ_*X*_: energy density of macronutrient *X* (kcal/g)
*FI*_*a*_: absorbed fat intake (g/day)
*CI*_*a*_: absorbed carbohydrate intake (g/day)
*FOx*_*net*_: [net fat oxidation] = [fat oxidation] – [*de novo* lipogenesis] (g/day) (50)
*COx*_*net*_: [net carbohydrate oxidation] = [carbohydrate oxidation] – [gluconeogenesis] + [*de novo* lipogenesis] (g/day) (50).

Before proceeding with the analysis, it is necessary to specify which variables in system s_1_ are treated as unknowns and which are treated as predetermined quantities. As shown in the Supplementary material, however, the resulting solution is independent of this choice. Accordingly, in the analysis that follows, the unknown variables in system s_1_ are *FI*_*a*_, *CI*_*a*_, and *PI*_*a*_, while all remaining terms are treated as determined quantities.

The number of unknown variables in system s_1_ can be reduced by noting that when weight stability and energy balance coincide, *FI*_*a*_ = *FOx*_*net*_. To demonstrate this, consider the identity:


$$\begin{array}{l} \frac{dFFM}{dt}=\frac{dBW}{dt}-\frac{dFM}{dt} \end{array}$$

Substituting this into the EBM governing equation (Equation 1) yields:


$$\begin{array}{l} EI-EE=\rho_{FM}\frac{dFM}{dt}+\rho_{FFM}\left(\frac{dBW}{dt}-\frac{dFM}{dt}\right)\\ \;=\left(\rho_{FM}-\rho_{FFM}\right)\frac{dFM}{dt}+\rho_{FFM}\frac{dBW}{dt}. \end{array}$$

Hence, under the standard EBM interpretation in which energy balance (*EI*−*EE* = 0) coincides with weight stability (*dBW*/*dt* = 0) the latter equation becomes


$$\begin{array}{l} \left(\rho_{FM}-\rho_{FFM}\right)\frac{dFM}{dt}=0. \end{array}$$

Finally, since ρ_*FM*_– ρ_*FFM*_ ≠ 0 (e.g., ρ_*FM*_– ρ_*FFM*_ = 9,300 – 1,100 = 8,200 kcal/kg (38)), it follows that *dFM*/*dt* = 0, which by Equation 4 implies that *FI*_*a*_ = *FOx*_*net*_.

As a result, all fat terms in s_1_ cancel out leading to


$$\begin{array}{l} \;CI_{a}+PI_{a}=COx_{net}+POx_{net}+OPL\\ \rho_{C}CI_{a}+\rho_{P}PI_{a}=\rho_{C}COx_{net}+\rho_{P}POx_{net}.\;\left(s_{2}\right) \end{array}$$

To clarify the analytical steps developed up to this point, the main results obtained so far are summarized below:

The coincidence of energy balance and weight stability leads to the condition *FI*_*a*_ = *FOx*_*net*_, thereby determining one of the unknown variables and allowing the original system s_1_ to be reduced to system s_2_. Solving s_2_ therefore also solves the constrained form of system s_1_, since the complete solution is recovered by reintroducing the imposed identity after solving the reduced system. Thus, the solution to s_1_, which summarizes the EBM requirements for the coincidence of weight stability (*dBW*/*dt* = 0) and energy balance (*EI* = *EE*), is


$$\begin{array}{l} FI_{a}=FOx_{net},\\ CI_{a}=x_{1},\\ PI_{a}=x_{2} \end{array}$$

where *x*_1_ and *x*_2_ are obtained by solving system s_2_.

As previously explained, the solution to system s_2_ must take into consideration that, at the coincidence of energy balance and weight stability, protein balance is zero (*PB* = 0), such that


$$\begin{array}{l} OPL=PI_{a}-POx_{net} \end{array}$$

Substituting this condition directly into system s_2_ yields the following solution (see Supplementary material):


$$\begin{array}{l} CI_{a}=x_{1}=COx_{net},\\ PI_{a}=x_{2}=POx_{net}. \end{array}$$

Substitution of *x*_2_ into the *OPL* equation gives


$$\begin{array}{l} OPL=PI_{a}-POx_{net}=POx_{net}-POx_{net}=0. \end{array}$$

This, however, represents an inconsistent physiological state, since *OPL* can never equal zero (56–58). Consequently, the condition *PB* = 0 at the coincidence of energy balance and weight stability cannot be imposed prior to solving system s_2_. Instead, it must be evaluated after obtaining the solution, since enforcing it beforehand leads to an internally inconsistent physiological condition (i.e., *OPL* = 0).

Although Atwater-based approximations often simplify the analysis by treating ρ_C_ ≈ ρ_P_, metabolic calorimetry studies (59–61) indicate that ρ_C_ ≠ ρ_P_ (e.g., ρ_*C*_≈ 4.2 kcal/g, ρ_*P*_≈ 4.7 kcal/g (61)). Indeed, this inequality is necessary to preserve physiological consistency because if ρ_C_ = ρ_P_, all solutions to system s_2_ require that *OPL* = 0, which would represent an unattainable physiological state (see Supplementary material).

Given that ρ_C_ ≠ ρ_P_, system s_1_ can be rewritten in matrix form


$$\begin{array}{l} \left[\begin{array}{cc} 1 & 1\\ \rho_{C} & \rho_{P} \end{array}\right]\left[\begin{array}{c} CI_{a}\\ PI_{a} \end{array}\right]=\left\{\begin{array}{c} COx_{net}+POx_{net}+OPL\\ \rho_{C}COx_{net}+\rho_{P}POx_{net} \end{array}\right\} \end{array}$$

and, after matrix inversion, yields the following solution:


$$\begin{array}{l} \left[\begin{array}{c} CI_{a}\\ PI_{a} \end{array}\right]=\frac{1}{\rho_{P}-\rho_{C}}\left[\begin{array}{cc} \rho_{P} & -1\\ -\rho_{C} & 1 \end{array}\right]\left\{\begin{array}{c} COx_{net}+POx_{net}+OPL\\ \rho_{C}COx_{net}+\rho_{P}POx_{net} \end{array}\right\}\\ \;=\left[\begin{array}{c} COx_{net}+\frac{\rho_{P}}{\rho_{P}-\rho_{C}}OPL\\ POx_{net}-\frac{\rho_{C}}{\rho_{P}-\rho_{C}}OPL \end{array}\right]. \end{array}$$

Therefore, at the coincidence of body weight stability and energy balance, the effective solution to system s_1_ implied by the accounting relationships of the EBM is:


$$\begin{array}{l} FI_{a}=FOx_{net},\\ CI_{a}=x_{1}=COx_{net}+\frac{\rho_{P}}{\rho_{P}-\rho_{C}}OPL,\\ PI_{a}=x_{2}=POx_{net}-\frac{\rho_{C}}{\rho_{P}-\rho_{C}}OPL. \end{array}$$

For this result to remain physiologically consistent with weight stability, the s_1_ solution must also yield zero protein balance. However, substituting *x*_2_ into the protein balance equation yields a persistent negative protein balance


$$\begin{array}{l} PB=PI_{a}-POx_{net}-OPL\\ \;=POx_{net}-\frac{\rho_{C}}{\rho_{P}-\rho_{C}}OPL-POx_{net}-OPL\\ \;=\frac{\rho_{P}}{\rho_{C}-\rho_{P}}OPL<0 \end{array}$$

since ρ_P_ > ρ_C_.

This result indicates that, according to the accounting relationships of the EBM, body mass is at the same time stable (because the solution to system s_1_ simultaneously satisfies both mass inflow = mass outflow and *EI* = *EE*, see Supplementary material) and persistently declining, since all absorbed protein is allocated to energy production through oxidation or gluconeogenesis while obligatory protein losses remain uncompensated (see Supplementary material). Consequently, the mathematical solution obtained by imposing the coincidence of energy balance and body weight stability is incompatible with the physiological requirement that long-term protein balance must be zero during true weight stability. Importantly, this conclusion should not be interpreted as a description of actual human physiology. Rather, it demonstrates that the simultaneous imposition of energy balance and body weight stability within the EBM accounting framework generates a logical inconsistency, as previously demonstrated (41).

## 3 Discussion

The present study examines the quantitative and logical coherence of the EBM when applied to controlled metabolic-ward datasets and to the accounting relationships that define the framework itself. The empirical analyses indicate that the quantitative correspondence expected between measured energy imbalance and observed body mass change is weaker than anticipated under highly controlled experimental conditions. In addition, the theoretical analysis presented in Section 2.9 shows that when the standard interpretation of the EBM under weight-maintenance conditions is imposed, the accounting relationships of the framework lead to an internally inconsistent outcome in which body mass is simultaneously stable and decreasing. Taken together, these findings suggest that the quantitative implementation of the EBM may exhibit less internal coherence and predictive robustness than generally assumed, even under highly controlled experimental conditions. The following discussion examines these results and their implications for interpreting experimental data on diet composition and body weight regulation.

### 3.1 Experimental uncertainty cannot fully explain the observed discrepancies

One potential explanation for the discrepancies identified in the numerical consistency tests is experimental uncertainty. Measurements of energy intake, energy expenditure, and body composition are subject to methodological error, and the interpretation of any quantitative comparison must therefore consider plausible measurement uncertainty.

The metabolic-ward trials examined in this manuscript were designed specifically to minimize such uncertainties. Energy intake and energy expenditure were measured using state-of-the-art methods, body composition was assessed using dual-energy X-ray absorptiometry, and dietary adherence was maintained under full inpatient supervision. These conditions represent some of the most controlled experimental environments available in human nutrition research. As discussed in Section 2.4, the discrepancies between independent estimates of energy balance in the first numerical test fall within plausible combined measurement uncertainty when propagated across all relevant measurements. Accordingly, that test alone does not constitute decisive evidence against the EBM.

This conclusion, however, does not readily extend to the remaining numerical consistency tests. In particular, the predicted directional relationships between energy imbalance and weight change were not consistently observed under controlled conditions. These analyses therefore reveal discrepancies that are difficult to reconcile with the quantitative relationships implied by the model. In the datasets analyzed here, relative prediction errors varied substantially across conditions, ranging from approximately 20 percent to nearly 400 percent depending on the experimental phase and the specific quantitative relationship examined. While measurement uncertainty undoubtedly contributes to some of this variation, discrepancies of this magnitude raise questions regarding the quantitative predictive capacity of the model when applied to controlled experimental interventions.

A common explanation for discrepancies between predicted and observed weight change in the energy balance literature is adaptive thermogenesis, whereby energy expenditure changes in response to dietary interventions (35–37). The present analysis does not dispute the existence of such physiological adaptations. Indeed, adaptive thermogenesis can modify the magnitude of energy expenditure and therefore alter the numerical value of energy imbalance during an intervention (35, 37). However, the numerical consistency tests developed here address a different question: whether the independently measured quantities that define energy balance remain mutually compatible within the accounting framework of the EBM. In other words, even if energy expenditure adapts physiologically, the measured values of intake, expenditure, body composition change, and macronutrient fluxes must still satisfy the accounting relationships of the model simultaneously. The present findings indicate that this condition is not always satisfied under controlled experimental conditions. Consequently, although adaptive thermogenesis may contribute to prediction error in specific experiments, it does not resolve the numerical inconsistencies identified in the present analysis. Beyond issues of experimental uncertainty and adaptive thermogenesis, the empirical discrepancies and the internal consistency analysis represent conceptually distinct lines of evidence. The former concerns the quantitative predictive performance of the EBM under controlled experimental conditions, whereas the latter examines whether the accounting relationships of the framework remain internally consistent when weight stability and energy balance are analytically imposed.

In other areas of quantitative physiology and biophysics, predictive models are typically expected to reproduce experimental measurements within relatively narrow error margins once key parameters are constrained. Models such as the classical Hodgkin-Huxley description of neuronal membrane dynamics achieve predictive errors on the order of only a few percent when evaluated against experimental data (4). By comparison, the larger discrepancies observed here suggest that the EBM may not function as a quantitatively precise predictive model of body mass dynamics under the conditions examined. Moreover, the present critique does not rely solely on empirical discrepancies. The internal consistency analysis presented in Section 2.9 further indicates that the accounting structure of the EBM generates a physiological contradiction when the coincidence of weight stability and energy balance is analytically imposed (i.e., body mass is simultaneously stable and decreasing). The mechanistic basis underlying this contradiction is discussed in the following section.

### 3.2 The internal inconsistency arises from equating energy balance with body mass balance

The preceding section showed that the critique developed here involves not only empirical discrepancies in predictive performance but also an internal inconsistency within the accounting structure of the EBM. The present section examines the mechanistic origin of that inconsistency and explains why it emerges when energy balance and body weight stability are treated as equivalent conditions within the framework.

The analysis presented here differs from previous discussions of the energy balance framework in that it evaluates not only empirical prediction error but also the internal accounting structure of the model itself. Whereas, earlier critiques have largely focused on measurement uncertainty or physiological adaptations (35–37), the present work examines whether the mathematical relationships that define the framework remain logically consistent when applied to the macronutrient fluxes that generate energy expenditure. Specifically, the mathematical analysis presented in Section 2.9 demonstrates that when energy balance (*EI* = *EE*) and weight stability are imposed simultaneously within the accounting structure of the EBM, the model requires that absorbed dietary protein be fully allocated to energy production through oxidation or gluconeogenesis, thereby leaving no absorbed protein available to compensate for obligatory protein losses (see Supplementary material). Because obligatory protein loss continues even in the absence of net energy imbalance, the model therefore predicts a persistent negative protein balance despite the assumption of body weight stability. This outcome implies an internally inconsistent physiological state in which body mass remains constant while protein mass is continuously lost. Importantly, the contradiction arises not from experimental data but from the internal accounting relationships of the model when energy balance and weight stability are treated as equivalent conditions.

The origin of this inconsistency lies in the conflation of energy dynamics with material dynamics. In any open physical system, mass balance and energy balance represent distinct conservation principles that govern different aspects of system behavior (54, 55). Energy flux describes the transfer of energy through the system, whereas mass balance describes the movement of matter. These two quantities are related but not interchangeable. In biological systems, body mass is determined by the balance between the mass of atoms entering the body through feeding and drinking and the mass of atoms leaving through oxidation products, excretion, and other physiological processes (62–64). Oxidizing one gram of macronutrient releases energy according to its specific energy density, but body mass decreases by one gram only when the oxidation products leave the body. Conversely, absorbing one gram of nutrient increases body mass by one gram regardless of its caloric value. By interpreting energy imbalance as directly governing body mass change, the EBM implicitly equates energy balance with mass balance. The analysis presented here indicates, consistent with the mathematical proof previously reported by Arencibia-Albite (41), that enforcing this equivalence within the accounting structure of the model produces an internal contradiction. This theoretical result complements the empirical findings of the numerical consistency tests and suggests that the limitations of the EBM arise from the structure of the framework itself rather than solely from experimental uncertainty.

### 3.3 . Reconsidering the operational definition of appetite in dietary intervention studies

The presence of internal inconsistency within the EBM has implications that extend beyond the evaluation of its numerical predictions. If a theoretical framework contains mutually incompatible assumptions, the interpretation of empirical data within that framework becomes ambiguous. In classical logic, a system that permits contradictory conclusions cannot reliably discriminate between competing explanations, because incompatible interpretations may appear equally valid within an inconsistent structure (65, 66). This possibility is relevant to the long-standing debate between the EBM and the carbohydrate-insulin model (CIM).

Within this context, the CIM offers a mechanistic hypothesis: under low carbohydrate diets, reduced postprandial insulin secretion may shift metabolism toward greater fat oxidation and potentially suppress appetite, mechanisms that could lead to weight and fat loss (67, 68). The metabolic ward experiment conducted by Hall et al. (39) was designed in part to evaluate these predictions under highly controlled conditions. In the original analysis of that study, appetite was operationally defined in terms of energy intake. Total food mass intake was recorded but was not incorporated into the appetite analysis. The published interpretation concluded that the LF diet produced lower energy intake than the LC diet and therefore appeared to suppress appetite more strongly (39).

However, when the results are examined alongside the observed changes in body composition and the measured patterns of food mass intake, this interpretation becomes less straightforward. During the initial phase of the trial, both diets produced statistically similar energy intake, energy expenditure, physical activity levels, and body mass energy density (see Figures 2A–E). Despite these similarities, only the LC diet produced a measurable reduction in fat mass, whereas the LF diet produced no comparable change (see Figures 4B_2_, C_2_).

An additional layer of complexity arises from endocrine measurements presented by Hall in a publicly available 2021 lecture at the George Washington University School of Medicine (69). Although these hormonal results were not included in the original publication, the reported patterns indicated that during LC feeding circulating concentrations of glucagon-like peptide-1 and peptide YY, hormones associated with appetite suppression, increased, whereas ghrelin, an appetite-stimulating hormone, decreased. During LF feeding the opposite pattern was observed.

If appetite suppression were accurately reflected by energy intake alone, the hormonal patterns would be expected to align with the reported energy intake results. However, during LC feeding, appetite-related hormonal signaling was consistent with reduced appetite despite the absence of lower energy intake relative to LF feeding. Across the phases of the study, LC feeding was nevertheless consistently associated with lower food mass intake, whereas LF feeding was not (Figure 6E). Taken together with the observed reductions in body and fat mass during LC feeding, these findings suggest that food mass intake may provide a more coherent behavioral representation of appetite than energy intake.

To understand why food mass intake may more closely reflect appetite across dietary interventions, it is necessary to consider how dietary energy density influences the relationship between energy intake and food mass intake. Dietary intervention studies fall into two broad categories: those comparing diets of similar energy density and those comparing diets that differ substantially in this regard. Because dietary energy density is the weighted average of the energy densities of its constituent macronutrients, weighted according to their mass fractions in the diet, diets with similar macronutrient composition tend to have similar energy density. Under these conditions, energy intake and food mass intake covary closely, so both measures may appear equally associated with appetite. However, this apparent agreement likely reflects the underlying dietary similarity rather than an independent link between appetite and energy intake per se. In contrast, when energy density differs substantially between interventions, the two measures diverge. In such cases, the present findings indicate that food mass intake remains more consistently aligned with appetite-related hormonal signaling and with observed changes in body composition. Overall, these observations suggest that food mass intake is the more consistent operational measure of appetite across dietary interventions, as it varies reliably with appetite-related hormonal signaling regardless of differences in energy density.

### 3.4 Toward an appetite-regulated mass balance perspective

The empirical numerical tests and the analytical consistency analysis presented above suggest that body weight dynamics may be more coherently described in terms of mass balance regulated by appetite, rather than purely in terms of energy balance. From a physical standpoint, changes in body mass must ultimately reflect changes in the quantity of matter contained within the body. Mass can change only when there is an imbalance between the mass entering the body through ingestion and the mass leaving the body through oxidation products, excretion, and other physiological processes (40, 41, 62–64). Energy balance, by contrast, describes the balance between energy inflow and outflow and does not by itself determine the quantity of matter contained within the system.

Within this perspective, diet composition can influence body weight indirectly by modifying physiological signals that regulate appetite and feeding behavior. Hormonal signals, substrate availability, and metabolic adaptations influence the internal cues that determine when eating begins and ends. These signals ultimately determine the quantity of food mass consumed before satiety is reached. When appetite decreases, mass intake falls and the resulting mass balance favors weight reduction. When appetite increases, mass intake rises and the resulting mass balance favors weight gain. Energy intake in this framework becomes a derived quantity, determined by the caloric density of the food mass consumed rather than functioning as the primary causal driver of body weight change.

The present findings therefore motivate consideration of an appetite-regulated mass balance model (ARMBM) in which appetite-mediated food mass intake represents the primary behavioral determinant of body weight dynamics under conditions of unrestricted feeding.

Importantly, the ARMBM is introduced here as a conceptual framework rather than as a fully parameterized predictive model. Although the present findings are compatible with this perspective, the ARMBM has not yet undergone extensive independent experimental validation and should therefore be regarded as a hypothesis-generating framework requiring further empirical evaluation. Historically, nutritional modeling has focused primarily on energy accounting (8–24), and relatively little work has explored body weight dynamics explicitly from the standpoint of mass conservation under free-feeding conditions. To date, only limited formal explorations of mass balance models have been proposed (40, 41). The present analysis extends this earlier work by showing that incorporating appetite regulation and diet-induced metabolic adaptation may provide a more coherent interpretation of observed feeding behavior and body weight outcomes. Notably, the critique of the EBM presented in this article does not depend on the completeness of ARMBM. Demonstrating that a model fails necessary consistency conditions does not require that a fully developed alternative already exist. The ARMBM is therefore presented primarily to illustrate that a physically coherent alternative framework is possible and to outline directions for future theoretical and experimental investigation.

### 3.5 Conclusion

The analyses presented in this study indicate that the EBM faces both empirical and theoretical challenges when examined as a quantitative model of body mass dynamics. The numerical consistency tests applied to controlled metabolic-ward datasets reveal discrepancies between predicted and observed outcomes that are difficult to reconcile solely through measurement uncertainty. In addition, the mathematical analysis presented in Section 2.9 shows that when the coincidence of energy balance and weight stability is imposed within the accounting structure of the EBM, the framework produces an internally inconsistent result in which body weight is mathematically stable while simultaneously declining through uncompensated obligatory protein loss. These findings suggest that the EBM may function more appropriately as a descriptive accounting convention than as a predictive biophysical model of body weight regulation. Reframing body weight dynamics in terms of appetite-regulated mass balance offers a conceptually coherent alternative that is consistent with fundamental conservation principles and with the behavioral regulation of feeding. Within such a framework, diet composition influences metabolic signaling, metabolic signaling influences appetite, and appetite determines the quantity of food mass consumed, thereby establishing the mass balance that ultimately governs changes in body weight.

The appetite-regulated mass balance model proposed here represents an initial conceptual step toward a modeling framework grounded explicitly in mass conservation and physiological regulation of feeding behavior. Further empirical and theoretical work will be necessary to develop this framework into a fully parameterized predictive model. Nevertheless, the present analysis suggests that such an approach may provide a more coherent basis for interpreting dietary interventions and for resolving long-standing debates in the field of human nutrition and metabolism.

## Data Availability

The data used in this study were obtained from the published results of Hall et al. (38) and from the Open Science Framework repository for Hall et al. (39) (https://osf.io/zdwqb/).
