# Supplementary material for: A numerical and analytical evaluation of the consistency of the energy balance model identifies limitations and systematic relationships between mass intake and body weight dynamics
Source: Front Nutr. 2026 Jul 30;13:1777554. doi: 10.3389/fnut.2026.1777554 (PMC13468908; doi:10.3389/fnut.2026.1777554)
Supplement: Supplementary file 1 [file Supplementary_file_1.docx]

**Supplementary material**

*Table S1*

| **Phase** | **Measured EB**  **(kcal/day)** | **Predicted ΔBW (kg)** | **Observed ΔBW (kg)** | **Required EB to reproduce observed ΔBW**  **(kcal/day)** | **Required correction to measured EB (kcal/day)** | **Interpretation** |
| --- | --- | --- | --- | --- | --- | --- |
| LC Initial | +38.5 | +0.078 | −2.809 | −1385 | −1423 | Direction and magnitude disagreement; requires very large correction |
| LF Initial | +231 | +0.498 | −0.647 | −300 | −531 | Direction disagreement; requires moderate correction |
| LC Final | +776.9 | +1.68 | −0.562 | −261 | −1038 | Direction and magnitude disagreement; requires very large correction |
| LF Final | −520.0 | −1.06 | −1.318 | −645 | −125 | Direction preserved; most compatible case |

EB: Energy Balance

*Table S2*

| **Phase** | **Predicted**  **ΔFM** | **Observed**  **ΔFM** | **No**  **DXA**  **correction** | **+0.5 kg**  **favorable DXA**  **correction** | **+1.0 kg**  **favorable DXA**  **correction** |
| --- | --- | --- | --- | --- | --- |
| LC Initial | +0.560 | −0.688 | FOx +59% | FOx +35% | FOx +12% |
| LF Initial | +0.035 | −0.424 | FOx +109% | Overlaps | Overlaps |
| LC Final | +2.198 | +0.562 | FOx +87% | FOx +61% | FOx +34% |
| LF Final | −0.560 | −0.890 | FOx +40% | Overlaps | Overlaps |

*Derivation of Equation 1: The EBM governing equation*

Total body energy (BE, in kcal) is the sum of the energy present in fat mass (FME) and fat-free mass (FFME):

$BE=FME+FFME$.

The energy content of each body compartment is equal to the product of its mass (kg) and energy density (kcal/kg). Hence,

$$FME=\rho_{FM}FM, FFME=\rho_{FFM}FFM,$$

where *FM* and *FFM* are the fat mass and fat-free mass, respectively, and *ρ_FM_* and *ρ_FFM_* are their corresponding energy densities, which are characteristic constants of body mass (20, 38).

Substituting into the first expression gives

$BE=\rho_{FM}FM+\rho_{FFM}FFM$.

Differentiating BE with respect to time *t* (in days) yields

$\frac{dBE}{dt}=\rho_{FM}\frac{dFM}{dt}+\rho_{FFM}\frac{dFFM}{dt}$.

By the principle of energy conservation, total body energy increases when energy intake (EI, in kcal/day) exceeds energy expenditure (EE, in kcal/day) and decreases when the opposite occurs. Thus,

$\frac{dBE}{dt}=EI-EE$.

Therefore, combining the previous two equations gives the EBM governing equation:

$EI-EE=\rho_{FM}\frac{dFM}{dt}+\rho_{FFM}\frac{dFFM}{dt}$.

*Derivation of Equation 2*

If *X* is a continuous variable that evolves over time, then its average value over an interval of length *T* is defined as

$\left\langle X\right\rangle=\frac{1}{T}\int_{0}^{T} X dt$.

Consequently, the average energy balance during an intervention lasting *T* days is

$\left\langle EI\right\rangle-\left\langle EE\right\rangle=\frac{1}{T}\int_{0}^{T} EI dt-\frac{1}{T}\int_{0}^{T} EE dt=\frac{1}{T}\int_{0}^{T} \left(EI-EE\right) dt$.

Integration of the above expression after substituting the integrand with Equation 1 yields Equation 2:

$$\begin{matrix} \left\langle EI\right\rangle-\left\langle EE\right\rangle=\frac{1}{T}\int_{0}^{T} \left(\rho_{FM}\frac{dFM}{dt}+\rho_{FFM}\frac{dFFM}{dt}\right) dt \\ =\frac{ \rho_{FM}\int_{0}^{T} \frac{dFM}{dt} dt + \rho_{FFM}\int_{0}^{T} \frac{dFFM}{dt} dt }{T} \\ =\frac{ \rho_{FM}\int_{FM_{initial}}^{FM_{final}} dFM + \rho_{FFM}\int_{FFM_{initial}}^{FFM_{final}} dFFM }{T} \\ =\frac{ \rho_{FM}\left(FM_{final}-FM_{initial}\right) + \rho_{FFM}\left(FFM_{final}-FFM_{initial}\right)}{T} \\ =\frac{ \rho_{FM}\Delta FM + \rho_{FFM}\Delta FFM}{T}. \end{matrix}$$

*Derivation of the body composition equations*

Body weight (*BW*) is the sum of fat mass (*FM*) and fat-free mass (*FFM*):

$$BW=FM+FFM.$$

Differentiation with respect to time *t* (in days) yields:

$\frac{dBW}{dt}=\frac{dFM}{dt}+\frac{dFFM}{dt}$.

By the chain rule

$$\frac{dBW}{dt}=\frac{dFM}{dt} + \frac{dFFM}{dFM}\frac{dFM}{dt}=\left[ 1 + \frac{dFFM}{dFM}\right]\frac{dFM}{dt}.$$

Solving for *dFM/dt*

$\frac{dFM}{dt}=\left[\frac{1}{1 + dFFM/dFM}\right]\frac{dBW}{dt}$.

Also,

$$\begin{matrix} \frac{dFFM}{dt}=\frac{dBW}{dt} - \frac{dFM}{dt} \\ =\frac{dBW}{dt} - \left[\frac{1}{1 + dFFM/dFM}\right]\frac{dBW}{dt} \\ =\left[1 - \frac{1}{1 + dFFM/dFM}\right]\frac{dBW}{dt} \\ =\left[\frac{dFFM/dFM}{1 + dFFM/dFM} \right]\frac{dBW}{dt}. \end{matrix}$$

Therefore, the temporal evolution of body composition can be computed as:

$$\begin{matrix} \frac{dFM}{dt}=\left[\frac{1}{1 + dFFM/dFM}\right]\frac{dBW}{dt}, \\ \\ \frac{dFFM}{dt}=\left[\frac{dFFM/dFM}{1 + dFFM/dFM}\right]\frac{dBW}{dt}. \end{matrix}$$

*Derivation of Equation 3: The second version of EBM governing equation*

Substitution of the body composition equations into Equation 1 yields

$$\begin{matrix} EI - EE=\rho_{FM}\frac{dFM}{dt} + \rho_{FFM}\frac{dFFM}{dt} \\ =\rho_{FM}\left[\frac{1}{1 + dFFM/dFM}\right]\frac{dBW}{dt} + \rho_{FFM}\left[\frac{dFFM/dFM}{1 + dFFM/dFM}\right]\frac{dBW}{dt} \\ =\left[\frac{ \rho_{FM} + \rho_{FFM}dFFM/dFM}{1 + dFFM/dFM }\right]\frac{dBW}{dt} \end{matrix}$$

where the expression within the squared brackets computes the body mass energy density (*ρ_BM_*):

$\rho_{BM}=\frac{ \rho_{FM} + \rho_{FFM}dFFM/dFM}{1 + dFFM/dFM }$.

Hence,

$EI - EE=\rho_{BM}\frac{dBW}{dt}$.

Solving for *dBW/dt* produces Equation 3:

$\frac{dBW}{dt}=\frac{EI - EE}{\rho_{BM}}$.

*Derivation of Equation 4*

According to the energy balance framework, fat accumulates through the combined action of absorbed fat intake (*FI_a_*, g/day) and de novo lipogenesis (*DNL*, g/day):

Fat Acumulation = *FI_a_* + *DNL.*

In contrast, fat loss results from fat oxidation (*FOx*, g/day):

Fat Loss = *FOx.*

Therefore, the daily rate of fat mass change is given by

$$\begin{matrix} \frac{dFM}{dt}=\left(\text{Fat Acumulation}\right)-\left(\text{Fat Loss}\right) \\ =FI_{a}+DNL-FOx \\ =FI_{a}-\left(FOx-DNL\right). \end{matrix}$$

Since net fat oxidation (*FOx_net_*) is defined as

*FOx_net_* = *FOx* – *DNL,*

the above expression can be rewritten as

$$\frac{dFM}{dt}=FI_{a}-FOx_{net}$$

which is Equation 4.

*Derivation of Equation 5*

The change in fat mass (Δ*FM*) during an intervention lasting *T* days is

$\Delta FM=FM_{final}-FM_{initial}=\int_{FM_{initial}}^{FM_{final}} dFM=\int_{0}^{T} \frac{dFM}{dt}dt$.

Substituting the integrand by Equation 4 gives

$\Delta FM=\int_{0}^{T} \left(FI_{a}-FOx_{net}\right)dt$.

Hence, the average fat balance during the intervention is

$$\begin{matrix} \left\langle FI_{a}\right\rangle-\left\langle FOx_{net}\right\rangle=\frac{1}{T}\int_{0}^{T} FI_{a} dt-\frac{1}{T}\int_{0}^{T} FOx_{net} dt \\ =\frac{1}{T}\int_{0}^{T} \left(FI_{a}-FOx_{net}\right) dt=\frac{\Delta FM}{T}. \end{matrix}$$

Therefore, solving Δ*FM* gives Equation 5:

$\Delta FM=\left[\left\langle FI\right\rangle-\left\langle FOx_{net}\right\rangle\right]\cdot T$.

where *FI_a_* has been replaced by fat intake (*FI*), because in the empirical application of Equation 4 the difference between *FI_a_* and measured *FI* is negligible (49).

*Derivation of Equation 6*

The change in body weight (Δ*BW*) during an intervention lasting *T* days is

$$\Delta BW=BW_{final}-BW_{initial}=\int_{BW_{initial}}^{BW_{final}} dBW=\int_{0}^{T} \frac{dBW}{dt}dt.$$

If *ρ_BM_* remains constant over *T*, then substituting the integrand by Equation 3 gives

$\Delta BW=\frac{1}{\rho_{BM}}\int_{0}^{T} \left(EI-EE\right)dt$.

Dividing both sides by *T*

$\frac{\Delta BW}{T}=\frac{1}{\rho_{BM}}\left[\frac{1}{T}\int_{0}^{T} \left(EI-EE\right)dt\right]$.

Since the average energy balance during an intervention is defined as

$$\left\langle EI\right\rangle-\left\langle EE\right\rangle=\frac{1}{T}\int_{0}^{T} \left(EI-EE\right) dt$$

the above expression can be rewritten as

$$\Delta BW=\frac{\left[\left\langle EI\right\rangle-\left\langle EE\right\rangle\right]\cdot T}{\rho_{BM}}$$

which is Equation 6.

*Solution to s_2_ with OPL = PI_a_ – POx_net_*

If the protein balance is zero (*PB* = 0), then

*OPL = PI_a_ – POx_net_*.

Substituting this condition into the mass balance equation of system s_2_ yields

$$\begin{matrix} CI_{a} + PI_{a}=COx_{net} + POx_{net}+ \overbrace{\left( PI_{a}-POx_{net}\right)}^{= OPL} \\ ⇓ \\ CI_{a} =COx_{net}. \end{matrix}$$

Substituting this result into the energy balance equation of s_2_ gives

$\begin{matrix} \rho_{C}\overbrace{COx_{net}}^{= CI_{a}} + \rho_{P}PI_{a}=\rho_{C}COx_{net} + \rho_{P}POx_{net} \\ ⇓ \\ PI_{a}= POx_{net}. \end{matrix}$.

Therefore, the solution to system s_2_ under the condition *OPL = PI_a_ – POx_net_* is

$$\begin{matrix} CI_{a} =COx_{net}, \\ PI_{a}= POx_{net}. \end{matrix}$$

*Solution to s_2_ by matrix inversion* (*ρ_P_* ≠ *ρ_C_*)

*Independence of the s_1_ solution from the choice of unknown variables*

To prove that the solution to s_1_ is independent of the particular variables treated as unknowns, it is sufficient to solve system s_2_ under an alternative choice of unknown quantities. Specifically, consider the case in which *COx_net_* and *POx_net_* are treated as the unknown variables.

Under this choice, system s_2_ can be rewritten as

$$\begin{matrix} COx_{net} + POx_{net}=CI_{a} + PI_{a}-OPL \\ \rho_{C}COx_{net} + \rho_{P} POx_{net}=\rho_{C}CI_{a} + \rho_{P}PI_{a} \end{matrix}$$

or, in matrix form,

$\left[\begin{matrix} 1 & 1 \\ \rho_{C} & \rho_{P} \end{matrix}\right]\left[\begin{matrix} COx_{net} \\ POx_{net} \end{matrix}\right]=\left\{\begin{matrix} CI_{a} + PI_{a}-OPL \\ \rho_{C}CI_{a} + \rho_{P}PI_{a} \end{matrix}\right\}$.

Solving by matrix inversion gives

.

Thus, the resulting solution to s_1_ becomes

which is identical to the solution previously obtained when the absorbed macronutrient fluxes were treated as the unknown variables. Therefore, the solution to s_1_ is invariant with respect to the particular choice of unknown quantities, completing the demonstration.

*If ρ_C_ = ρ_P_ then OPL=0*

Proof: If *ρ_C_* = *ρ_P_*, then s_2_ becomes

$$\begin{matrix} CI_{a} + PI_{a}=COx_{net} + POx_{net}+ OPL \\ CI_{a} + PI_{a}= COx_{net} + POx_{net} \end{matrix}$$

Subtracting the second equation from the first yields

$$OPL=0$$

which completes the proof.

*The solution of system s_1_ guarantees the coincidence of mass balance and energy balance*

The solution of system s_1_ is:

To verify that this solution indeed yields the coincidence of mass balance and energy balance, it is sufficient to demonstrate that substituting the solution into the mass inflow equation reduces it to the mass outflow equation:

Similarly, substitution of the solution into the energy intake equation transforms the resulting expression into the energy expenditure equation:

Thus, the solution to system s_1_ indeed generates the simultaneous conditions of mass inflow = mass outflow and energy intake = energy expenditure.

*Why the solution to system s_1_ implies that all absorbed protein is allocated to energy production while obligatory protein losses remain uncompensated*

True body weight stability requires that *PB* = 0, implying that

$$PI_{a}=POx_{net}+OPL.$$

Thus, under steady body weight, absorbed dietary protein is expected to compensate for both oxidative protein loss (*POx_net_*) and obligatory protein losses (*OPL*).

However, the protein component of solution to system s_1_ is

where *ρ_P_* > *ρ_C_* and *OPL* > 0.

Thus, according to the EBM, at the coincidence of energy balance and weight stability, the required rate of net protein oxidation exceeds the rate of dietary protein absorption by

Consequently, the absorbed dietary protein supply (*PI_a_*) is entirely allocated to energy production through direct oxidation or gluconeogenesis followed by oxidation. The additional protein required to satisfy the oxidative demand,

must therefore be drawn from endogenous protein stores. Furthermore, because all absorbed dietary protein is committed to energy production, none remains available to replace obligatory protein losses.

In conclusion, dietary protein fails to fulfill its expected role under true body weight stability, namely, compensating for both oxidative protein losses and obligatory protein losses. As a result, obligatory protein losses remain uncompensated, producing a negative protein balance despite the fact that the solution to system s_1_ simultaneously satisfies the conditions of energy balance and body weight stability (see previous section).
